# Fractal perspective of superquadratic functions with generalized probability estimations

**DOI:** 10.1371/journal.pone.0313361

**Published:** 2025-02-11

**Authors:** Saad Ihsan Butt, Dawood Khan, Youngsoo Seol

**Affiliations:** 1 Department of Mathematics, COMSATS University Islamabad, Islamabad, Pakistan; 2 Department of Mathematics, Dong-A University, Busan, Korea; Universiti Tun Hussein Onn Malaysia, MALAYSIA

## Abstract

This study introduces for the first time a class of generalized superquadratic functions specifically on fractal sets and explores their unique features. The research develops several generalized inequalities, including Jensen’s, converse Jensen’s, Mercer Jensen’s and Hermite-Hadamard’s inequalities based on the properties of generalized superquadratic functions. The findings are confirmed through reduced results, numerical calculations and graphical depictions, ensuring the robustness and accuracy of the proposed inequalities by taking into account several appropriate examples. A detailed comparative analysis between inequalities derived from generalized superquadratic functions and those from generalized convex functions, highlighting the greater refinement provided by the generalized superquadratic functions. The study enhances its findings with practical applications in probability expectations and special means in fractal space, demonstrating the applicability and relevance of the new results in these domains. The new results presented in this work provide significant extensions and improvements over existing literature, showcasing advancements and potential for further research in the field.

## Introduction

Mathematical inequalities are regarded as the main framework for gathering the quantitative and qualitative interpretation in the applied sciences. It is commonly recognized that inequalities have played a significant role in the development and expansion of mathematics. Convex functions have a great role in many disciplines, including coding hypothesis, optimisation, material science, data hypothesis, designing, and inequality theory. They also have potential applications in many fascinating and challenging fields of study. As a result, mathematicians consistently invest their potential and labour in this field to explore and unearth a wide range of conclusions that are important and useful for applications. In science and engineering, inequalities have recently seen extraordinary theoretical and practical development [1–6]. One of the primary uses of the convex function is the construction of inequalities. In many different disciplines, excellent inequalities of different classes identified with convex function have been built and put to use.

The idea of one-variable superquadratic function as a generalisation of a class of convex function was initiated by Abramovich et al. in [7]. A superquadratic function f(ϰ):I⊆[0,∞)→R, must meets the condition (1), ∀ y_**o**_, *ϰ* ≥ 0 and $C_{\varkappa }\in R$.

$$\begin{array}{c} f\left({\text{y}}_{\text{o}}\right)\geq f\left(\varkappa \right)+C_{\varkappa }\left({\text{y}}_{\text{o}}-\varkappa \right)+f(|{\text{y}}_{\text{o}}-\varkappa \left|\right), \end{array}$$ (1)

where $C_{\varkappa }$ is termed as a constant.

The terms superquadraticity and subquadraticity have the same role as that of convexity and concavity of a function respectively such that given one we can determine the next one. For example if f is superquadratic then subquadratic function is obtained by -f. In the subsequent we will consider an example whose superquadraticity and subquadraticity does not depend on altering the sign of the function.

The function $f\left(\varkappa \right)=\varkappa^{p}$, for every *ϰ* ≥ 0, is superquadratic and subquadratic for p≥2 and 0≤p<2 respectively. In this scenario, $C_{\varkappa }=f^{\prime }\left(\varkappa \right)$. The consideration of p=2 in a function $f\left(\varkappa \right)=\varkappa^{p}$, replaces the sign “≤” by “=” in (1).

The fundamental properties of superquadratic functions have been explained in [7, 8] by the well known researchers Sinnamon, Jameson and Abramovich. The properties and integral inequalities based on the condition (1) are more refined as compared to the convexity. The conditions (i), (ii) and (iii) which are listed below must be fulfilled by any arbitrarily superquadratic function:

(*i*) f(0)≤0,
(*ii*) If f is differentiable at *ϰ* > 0 as well as f(0)=0 and $f^{\prime }\left(0\right)=0$ then $C_{\varkappa }=f^{\prime }\left(\varkappa \right)$,
(*iii*) f is convex and f(0)=0 as well as $f^{\prime }\left(0\right)=0$ if f≥0.

The two major inequalities that widen and extrapolate the concept of superquadraticity are the Hermite-Hadamard’s and Jensen’s integral inequalities. These two inequalities are the utmost important and extensively used results involving superquadratic functions. The classical Jensen’s inequality enlighten an essential characterization of superquadratic functions:

**Theorem 1** [8] *If* f *is assumed to be a superquadratic*. *Let* *ϰ*_*i*_ ≥ 0 *and* 0 ≤ λ_*i*_ ≤ 1, *and let* $\bar{\varkappa }=\Sigma_{i=1}^{n}\lambda_{i}\varkappa_{i}$, *where* λ_*i*_ ≥ 0 *and* $\Sigma_{i=1}^{n}$λ_*i*_ = 1. *Then*

$$\begin{array}{cc} & \Sigma_{i=1}^{n}\lambda_{i}f\left(\varkappa_{i}\right)\geq f\left(\bar{\varkappa }\right)+\Sigma_{i=1}^{n}\lambda_{i}f(|\varkappa_{i}-\bar{\varkappa }\left|\right). \end{array}$$

Inequalities of Hermite-Hadamard’s type in the field of superquadraticity was developed by Banić et al. in [9].

**Theorem 2** *Let* $I=[a_{o},b_{o}]\subseteq [0,\infty [$ *where* $0\leq a_{o}<b_{o}$ *and the mapping* f:I→R *is superquadratic then*

$$\begin{array}{cc} & f(\frac{a_{o}+b_{o}}{2})+\frac{1}{b_{o}-a_{o}}\int_{a_{o}}^{b_{o}}f(|\varkappa -\frac{a_{o}+b_{o}}{2}|)d\varkappa \leq \frac{1}{b_{o}-a_{o}}\int_{a_{o}}^{b_{o}}f\left(\varkappa \right)d\varkappa \\ & \leq \frac{f\left(a_{o}\right)+f\left(b_{o}\right)}{2}-\frac{1}{{\left(b_{o}-a_{o}\right)}^{2}}\int_{a_{o}}^{b_{o}}\left[\left(b_{o}-\varkappa \right)f\left(\varkappa -a_{o}\right)+\left(\varkappa -a_{o}\right)f\left(b_{o}-\varkappa \right)\right]d\varkappa . \end{array}$$

Superquadratic function can also be defined as the function which fulfils the condition (2), ∀ *ϰ*_1_, *ϰ*_2_ ≥ 0 and λ ∈ (0, 1).

$$\begin{array}{cc} & f\left(\left(1-\lambda \right)\varkappa_{1}+\lambda \varkappa_{2}\right)\leq \left(1-\lambda \right)f\left(\varkappa_{1}\right)+\lambda f\left(\varkappa_{2}\right)-\lambda f(\left(1-\lambda \right)|\varkappa_{1}-\varkappa_{2}\left|\right)\\ & -\left(1-\lambda \right)f(\lambda |\varkappa_{1}-\varkappa_{2}|), \end{array}$$ (2)

for nonnegative superquadratic function the condition (2) is termed as the refinement of the Jensen’s inequality. If the inequality in (2) is flipped then the function f behaves like subquadratic.

It deserves to be recalled that an additional set of superquadratic functions had been developed years earlier in [10] and was subject to discussion, for instance, in [11]. There are possibilities where the two descriptions coincide, but these are exceptional. The connections and distinctions between these two classes are explained in detail by Gilányi in [12]. In [13], Chesneau and Alomari suggested another sort of superquadratic functions identified as h-superquadratic functions, which broadens and improves the concept of superquadricity. Several of the essential features of h-superquadraticity were examined and expounded upon. Logarithmically superquadratic function is one of the essential type of superquadraticity which was pioneered by Mario Krnić in [14] and determined the necessary prerequisites for it. Khan and Butt are the first who originated the idea of interval valued cr-superquadratic functions in [15] as well as the inequalities of Hermite-Hadamard and Fejér types along with their fractional version in [16]. They not only determined the properties of superquadraticity in terms of center radius cr-order relation but also considered its fractional version for interval valued cr-superquadratic functions. Our effort in this study was motivated by what had previously indicated contribution to the field of superquadraticity, whereby we develop the new Jensen’s and Hermite-Hadamard’s types of inequalities for superquadratic functions and provide applications.

Readers who are interested in learning more about superquadraticity can turn to [17–19] and the sources given therein to gain knowledge about their numerous properties, examples, and potential applications in the context of inequality.

Kolwankar and Gangal [20] established the theory of local fractional calculus (sometimes identified by fractal calculus), which has gotten a close consideration for its use in non-differentiable problems in engineering and science. Fractal calculus is essential for modeling systems that exhibit fractal behavior, which traditional calculus cannot effectively describe. These systems are found in a wide range of natural phenomena, including coastlines, clouds, mountain ranges, and biological structures like blood vessels and neural networks. The non-integer dimensions and self-similar patterns of fractals require a mathematical approach that can handle such irregularities, which fractal calculus provides. Traditional calculus often falls short when dealing with irregular, fragmented, or self-similar structures that are characteristic of fractals. Fractal calculus allows for more accurate mathematical modeling of these structures, leading to better predictions and understanding of complex systems. Fractal calculus opens up new avenues for interdisciplinary research, bridging gaps between mathematics, physics, biology, economics, and more. It provides a framework for developing new theories and models that can be applied across various fields, leading to innovations in technology and scientific understanding. By using fractal calculus, scientists can gain deeper insights into natural phenomena that exhibit fractal properties. This enhanced understanding can lead to discoveries about the underlying principles governing complex systems, which might be overlooked by traditional approaches. Fractal calculus is increasingly important in the analysis of complex datasets, particularly those that display fractal characteristics. It allows for the development of new algorithms and techniques for analyzing data in fields such as finance, climate science, and network theory, where traditional methods may not be sufficient. In 2012, Yang [21], inspired by these applications, established a reflection of local fractional functions on fractal sets, which related to the local fractional calculus and function monotonicity. Local fractional calculus generalizes the differentiation and integration of functions defined on fractal sets. The concept of local fractional calculus has piqued the curiosity of mathematicians, as well as physicists and engineers. The theory of local fractional plays a vital role in a wide range of applications, including theoretical physics, elasticity and fracture mechanics theory [21–24], and so on. In (2017) Sun [25] developed the notion of generalized harmonic convex function on fractal sets $R^{\alpha }$ and displayed certain inequalities of Hermite-Hadamard’s type for this class. In (2020) Sun [26] proposed the *α*-type concept of generalized *h*-convex function on Yang’s fractal sets $R^{\alpha }$. Some recent development in this direction can be seen in the following articles [27–31].

## Preliminaries

We recall the theory of Yang’s fractal calculus. For 0 < *α* ≤ 1, we have the following *α*-type set of element set:

$Z^{\alpha }=\{0^{\alpha },\pm 1^{\alpha },\pm 2^{\alpha },\ldots ,\pm n^{\alpha },\ldots \}$ .

$Q^{\alpha }=\{{\text{m}}_{o}^{\alpha }=({\text{r}}_{o}/{\text{s}}_{o}{)}^{\alpha }:{\text{r}}_{o},{\text{s}}_{o}\in Z,{\text{s}}_{o}\neq 0\}$ .

$J^{\alpha }=\{{\text{m}}_{o}^{\alpha }\neq ({\text{r}}_{o}/{\text{s}}_{o}{)}^{\alpha }:{\text{r}}_{o},{\text{s}}_{o}\in Z,{\text{s}}_{o}\neq 0\}$ .

$R^{\alpha }=Q^{\alpha }$ ∪ $J^{\alpha }$.

If ${\text{r}}_{o}^{\alpha },{\text{s}}_{o}^{\alpha }$, ${\text{t}}_{o}^{\alpha }$ ∈ $R^{\alpha }$ then the following operations satisfy

(i) ${\text{r}}_{o}^{\alpha }+{\text{s}}_{o}^{\alpha }$ ∈ $R^{\alpha }$, ${\text{r}}_{o}^{\alpha }{\text{s}}_{o}^{\alpha }$ ∈ $R^{\alpha }$;

(ii) ${\text{r}}_{o}^{\alpha }+{\text{s}}_{o}^{\alpha }=s^{\alpha }+{\text{r}}_{o}^{\alpha }={\left({\text{r}}_{o}+{\text{s}}_{o}\right)}^{\alpha }={\left({\text{s}}_{o}+{\text{r}}_{o}\right)}^{\alpha }$;

(iii) ${\text{r}}_{o}^{\alpha }+\left({\text{s}}_{o}^{\alpha }+{\text{t}}_{o}^{\alpha }\right)={\left({\text{r}}_{o}+{\text{s}}_{o}\right)}^{\alpha }+{\text{t}}_{o}^{\alpha }$;

(iv) ${\text{r}}_{o}^{\alpha }s^{\alpha }={\text{s}}_{o}^{\alpha }{\text{r}}_{o}^{\alpha }={\left({\text{r}}_{o}{\text{s}}_{o}\right)}^{\alpha }={\left({\text{s}}_{o}{\text{r}}_{o}\right)}^{\alpha }$;

(v) ${\text{r}}_{o}^{\alpha }\left({\text{s}}_{o}^{\alpha }{\text{t}}_{o}^{\alpha }\right)=\left({\text{r}}_{o}^{\alpha }{\text{s}}_{o}^{\alpha }\right){\text{t}}_{o}^{\alpha }$;

(vi) ${\text{r}}_{o}^{\alpha }\left({\text{s}}_{o}^{\alpha }+{\text{t}}_{o}^{\alpha }\right)={\text{r}}_{o}^{\alpha }{\text{s}}_{o}^{\alpha }+{\text{r}}_{o}^{\alpha }{\text{t}}_{o}^{\alpha }$;

(vii) ${\text{r}}_{o}^{\alpha }+0^{\alpha }=0^{\alpha }+{\text{r}}_{o}^{\alpha }={\text{r}}_{o}^{\alpha }$, ${\text{r}}_{o}^{\alpha }1^{\alpha }=1^{\alpha }{\text{r}}_{o}^{\alpha }={\text{r}}_{o}^{\alpha }$.

For the purpose of demonstrating the local fractional calculus on $R^{\alpha }$, we start by addressing the idea of local fractional continuity which is given as follows:

**Definition 1** [21] *A non-differentiable mapping* $\text{f}:R\to R^{\alpha }$, *ϰ* → f(*ϰ*) *at ϰ*_0_ *is named local fractional continuous at ϰ*_0_, *if for all ϵ* > 0 *there exists δ* > 0 *such that*

$$\begin{array}{c} |\text{f}\left(\varkappa \right)-\text{f}\left(\varkappa_{0}\right)|<\epsilon^{\alpha }, \end{array}$$

*holds for* |*ϰ* − *ϰ*_0_| < *δ*, *for all ϵ*, δ∈R. *If* f(*ϰ*) *is local fractional continuous on the interval* $\left(a_{o},b_{o}\right)$ *then we write* $\text{f}\left(\varkappa \right)\in C_{\alpha }\left(a_{o},b_{o}\right)$.

**Definition 2** [21] *The local fractional derivative of* f(*ϰ*) *of order α at* *ϰ* = *ϰ*_0_ *is*

$$\begin{array}{c} {\text{f}}^{\left(\alpha \right)}\left(\varkappa_{0}\right)=\frac{d^{\alpha }\text{f}\left(\varkappa \right)}{d\varkappa^{\alpha }}{|}_{\varkappa =\varkappa_{0}}=\underset{\varkappa \to \varkappa_{0}}{\text{lim}}\frac{\Delta^{\alpha }\left(\text{f}\left(\varkappa \right)-\text{f}\left(\varkappa_{0}\right)\right)}{{\left(\varkappa -\varkappa_{0}\right)}^{\alpha }}, \end{array}$$

*where* Δ^*α*^(f(*ϰ*) − f(*ϰ*_0_)) ≅ Λ(1 + *α*)(f(*ϰ*) − f(*ϰ*_0_)).

**Definition 3** [21] *If* $\text{f}\in C_{\alpha }\left[a_{o},b_{o}\right]$, *then the local fractional integral of* f(*ϰ*) *of order α is*

Iaoboαf(ϰ)=1Γ1+α∫aobof(z)(dz)α=1Γ1+αlimΔz→0∑M-1s=0f(zs)(Δzs)α,

*where* Δ*z*_*s*_ = *z*_*s*+1_ − *z*_*s*_, Δ*z* = *max* {Δ*z*_*s*_|*s* = 1, 2, …, *M* − 1}, *and* [*z*_*s*_, *z*_*s*+1_], *s* = 0, 1,.., *M* − 1 *with* $z_{0}=a_{o}<z_{1}<\ldots <z_{i}<\ldots <z_{M-1}<z_{M}=b_{o}$ *is a partition of* $\left[a_{o},b_{o}\right]$.

*Here, it implies that* Iaoboαf(ϰ)=0 *if* $a_{o}=b_{o}$ *and* Iaoboαf(ϰ)=-Iboaoαf(ϰ) *if* $a_{o}<b_{o}$ . *If for any* $\varkappa \in [a_{o},b_{o}]$, *there exists* Iaoboαf(ϰ), *then we denoted by* $\text{f}\left(\varkappa \right)\in I_{\varkappa }^{\left(\alpha \right)}\left[a_{o},b_{o}\right]$.

We denote $\text{f}\left(\varkappa \right)\in I_{\varkappa }^{\left(\alpha \right)}\left[a_{o},b_{o}\right]$ if there exits Iboϰαf(ϰ) for any $\varkappa \in [a_{o},b_{o}]$.

Define the Mittag-Leffler function [21] on fractal sets $R^{\alpha }$ (0 < *α* ≤ 1) is given by

$$\begin{array}{c} E_{\alpha }\left(\varkappa^{\alpha }\right)=\sum_{k=0}^{\infty }\frac{\varkappa^{k\alpha }}{\Gamma (1+k\alpha )},\;\varkappa \in R. \end{array}$$

**Lemma 1** [21]

IaoboαEα(ϰα)=Eα(boα)-Eα(aoα).dαEα(τϰα)dϰα=τEα(τϰα),

*where τ is a constant*.

**Lemma 2** [21]

*(i) If* $\text{f}\left(\varkappa \right)=g^{\left(\alpha \right)}\left(\varkappa \right)\in C_{\alpha }\left[a_{o},b_{o}\right]$ , *then we have*
  Iaoboαf(ϰ)=g(bo)-g(ao).
*(ii) If* $\text{f}\left(\varkappa \right),g\left(\varkappa \right)\in D_{\alpha }\left[a_{o},b_{o}\right]$ *and* ${\text{f}}^{\left(\alpha \right)}\left(\varkappa \right)$ , $g^{\left(\alpha \right)}\left(\varkappa \right)\in C_{\alpha }\left[a_{o},b_{o}\right]$, *then we have*
  Iaoboαf(ϰ)g(α)(ϰ)=f(ϰ)g(ϰ)|aobo-Iaoboαf(α)(ϰ)g(ϰ).

**Lemma 3** [21]

$$\begin{array}{c} \frac{d^{\alpha }\varkappa^{\tau \alpha }}{d\varkappa^{\alpha }}=\frac{\Gamma (1+\tau \alpha )}{\Gamma (1+(\tau -1)\alpha )}\varkappa^{(\tau -1)\alpha }, \end{array}$$

*and*

$$\begin{array}{c} \frac{1}{\Gamma (1+\alpha )}\int_{a_{o}}^{b_{o}}\varkappa^{\tau \alpha }{\left(d\varkappa \right)}^{\alpha }=\frac{\Gamma (1+\tau \alpha )}{\Gamma (1+(\tau +1)\alpha )}\left({b_{o}}^{(\tau +1)\alpha }-{a_{o}}^{(\tau +1)\alpha }\right), \end{array}$$

*while* τ∈R.

The core objective of this study is to establish Jensen, converse Jensen, Mercer Jensen and Hermite-Hadamard inequalities on fractal space for generalized superquadratic function. The validity of newly obtained inequalities are confirmed numerically and graphically by taking some appropriate examples along with some useful applications.

## Generalized superquadratic functions

From an analytical perspective, we are left with the subsequent definition:

**Definition 4** *A function* $\text{f}:I\subseteq \left[0,\infty \right)\to R^{\alpha }$ (0 < *α* ≤ 1) *is said to be generalized superquadratic function of fractal dimension α on I, if the following inequality*

$$\begin{array}{c} \text{f}(\lambda a_{o}+\left(1-\lambda \right)b_{o})\leq \lambda^{\alpha }(\text{f}\left(a_{o}\right)-\text{f}\left(\left(1-\lambda \right)|a_{o}-b_{o}|\right))+{\left(1-\lambda \right)}^{\alpha }(\text{f}\left(b_{o}\right)-\text{f}\left(\lambda |a_{o}-b_{o}|\right))), \end{array}$$ (3)

*holds for any* $a_{o},b_{o}\in I$ *and* λ ∈ [0, 1].

**Remark 1** *If α* = 1, *Definition 4 simplifies to the definition of superquadratic function. If the inequality in* (3) *flips, then the function* f *is termed as generalized subquadratic*.

Here we consider some basic examples of generalized superquadratic function.

**Example 1** *Mittag-Leffler function on fractal sets* $R^{\alpha }$ (0 < *α* ≤ 1) *given by*

$$\begin{array}{c} E_{\alpha }\left(\varkappa^{\alpha }\right)=\sum_{k=0}^{\infty }\frac{\varkappa^{k\alpha }}{\Gamma (1+k\alpha )},\;\varkappa \in R. \end{array}$$

*is a generalized superquadratic function for all ϰ* ≥ 3.

**Fig 1:**
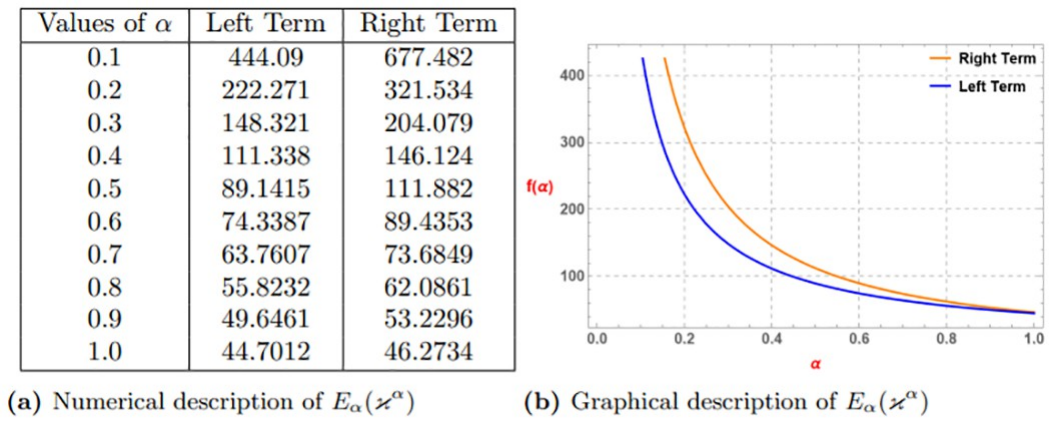
Numerical and graphical description of *E*_*α*_(*ϰ*^*α*^) via Definition 4. (**a**) Numerical description of *E*_*α*_(*ϰ*^*α*^). (**b**) Graphical description of *E*_*α*_(*ϰ*^*α*^).

*The subsequent Table 1a and Graph 1b given by* Fig 1, *confirm the validity of the aforementioned function to be generalized superquadratic function via Definition 4 for* $\lambda =0.2,a_{o}=3,b_{o}=4$ *and* (0 < *α* ≤ 1).

**Fig 2:**
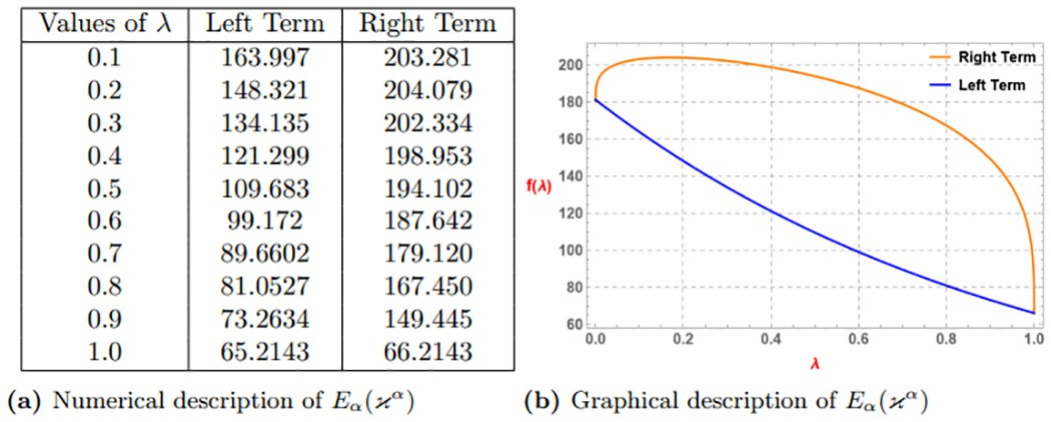
Numerical and graphical description of *E*_*α*_(*ϰ*^*α*^) via Definition 4. (**a**) Numerical description of *E*_*α*_(*ϰ*^*α*^). (**b**) Graphical description of *E*_*α*_(*ϰ*^*α*^).

*Similarly if we assume* $\alpha =0.3,a_{o}=3,b_{o}=4$ *and* (0 < λ ≤ 1) *then we get the subsequent Table 2a and Graph 2b given by* Fig 2 *which confirm the validity of the aforementioned function via Definition 4*.

**Example 2** *The following function is a generalized superquadratic function for all ϰ* ≥ 0 *and* p≥1.

$$\begin{array}{c} \text{f}\left(\varkappa \right)=-{\left(\varkappa +\text{k}\right)}^{-\alpha p},\forall \;\;\;\text{k}>0, \end{array}$$

*where* (0 < *α* ≤ 1).

**Fig 3:**
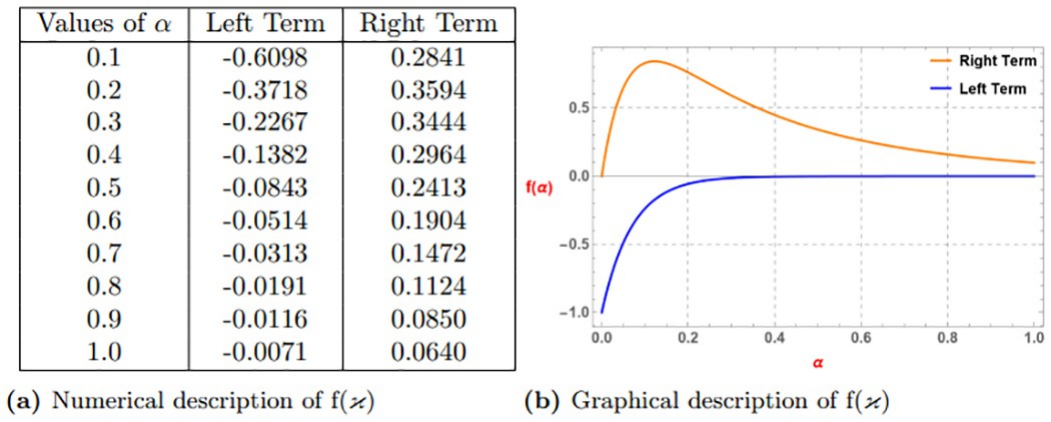
Numerical and graphical description of $\text{f}\left(\varkappa \right)=-{\left(\varkappa +\text{k}\right)}^{-\alpha p}$ satisfying Definition 4 for $\lambda =0.6,p=3,a_{o}=3,b_{o}=6,\text{k}=1$ and (0 < *α* ≤ 1). (**a**) Numerical description of f(*ϰ*). (**b**) Graphical description of f(*ϰ*).

*The subsequent Table 3a and Graph 3b given by* Fig 3, *confirm the validity of the aforementioned function via Definition 4 for* $\lambda =0.6,p=3,a_{o}=3,b_{o}=6,\text{k}=1$ *and* (0 < *α* ≤ 1).

**Fig 4:**
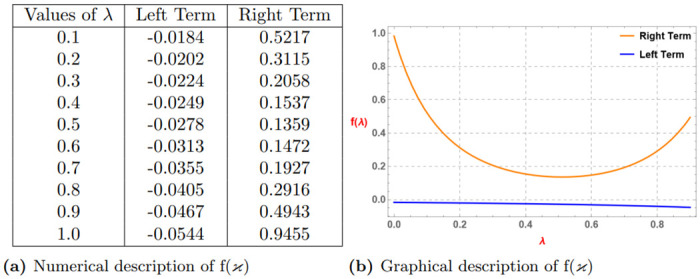
Numerical and graphical description of $\text{f}\left(\varkappa \right)=-{\left(\varkappa +\text{k}\right)}^{-\alpha p}$ satisfying Definition 4 for $\alpha =0.7,p=3,a_{o}=3,b_{o}=6,\text{k}=1$ and (0 < λ ≤ 1). (**a**) Numerical description of f(*ϰ*). (**b**) Graphical description of f(*ϰ*).

*Similarly, if we assume* $\alpha =0.7,p=3,a_{o}=3,b_{o}=6,\text{k}=1$ , *and* (0 < λ ≤ 1) *then we get The subsequent Table 4a and Graph 4b given by* Fig 4 *which confirm the validity of the aforementioned function via Definition 4*.

**Example 3** *The following function is a generalized superquadratic function for all ϰ* > 0 *and* p≥1.

$$\begin{array}{c} \text{f}\left(\varkappa \right)=\frac{\Gamma (1+\alpha p)}{1+\alpha (p+1)}\varkappa^{\alpha (p+1)}. \end{array}$$

**Fig 5:**
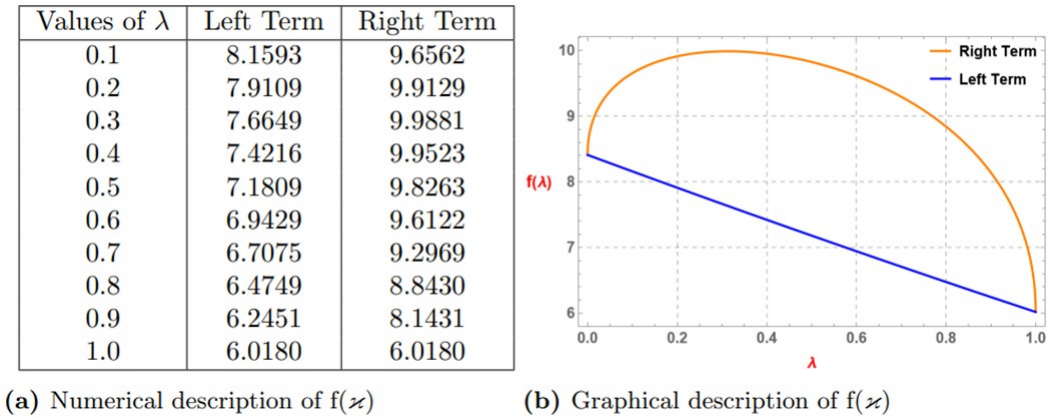
Numerical and graphical description of f(*ϰ*) satisfying Definition 4 for $\alpha =0.5,p=2,a_{o}=4,b_{o}=5$ and (0 < λ ≤ 1). (**a**) Numerical description of f(*ϰ*). (**b**) Graphical description of f(*ϰ*).

*The subsequent Table 5a and Graph 5b given by* Fig 5 *confirm the validity of the aforementioned function via Definition 4 for* $\alpha =0.5,p=2,a_{o}=4,b_{o}=5$ *and* (0 < λ ≤ 1).

**Fig 6:**
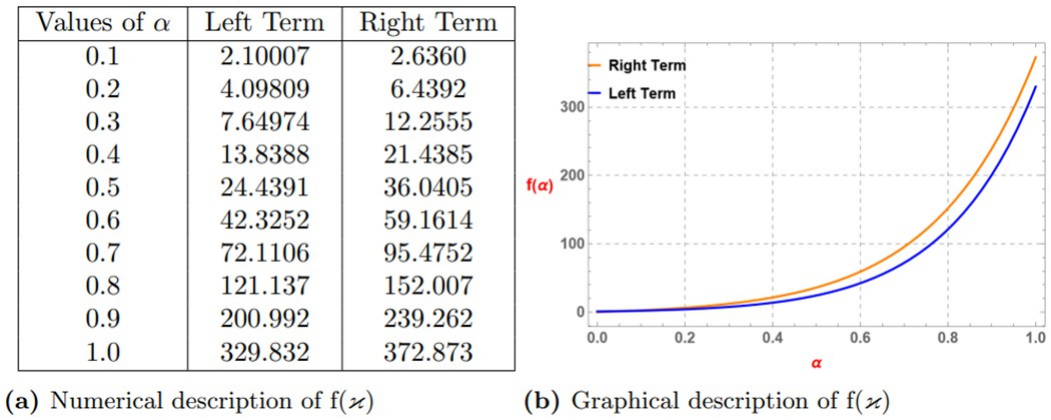
Numerical and graphical description of f(*ϰ*) satisfying Definition 4 for $\lambda =0.6,p=4,a_{o}=4,b_{o}=5$ and (0 < *α* ≤ 1). (**a**) Numerical description of f(*ϰ*). (**b**) Graphical description of f(*ϰ*).

*Similarly if we assume* $\lambda =0.6,p=4,a_{o}=4,b_{o}=5$ *and* (0 < *α* ≤ 1) *then we get the subsequent Table 6a and Graph 6b given by* Fig 6 *which confirm the validity of the aforementioned function via Definition 4*.

We shall direct our concentration on generalised superquadraticity, since a function f(*ϰ*) is generalized subquadratic only in the case ‘−f’ is a generalized superquadratic. Thus, every superquadratic findings may be simply reformulated in the context of subquadratic functions.

In the following, we will study the properties of the generalized superquadratic functions.

**Proposition 1** *If the functions* f *and* g *are assumed as generalized superquadratic then both* f+g *and* Kf , *where* K > 0 *are generalized superquadratic*.

***proof***: *Let* f *and* g *are generalized superquadratic functions i.e*., $\forall \;\;\;a_{o},b_{o}\in I,\lambda \in \left(0,1\right)$ *and* 0 < *α* ≤ 1 *we have*

$$\begin{array}{c} f(\lambda a_{o}+\left(1-\lambda \right)b_{o})\leq \lambda^{\alpha }(f\left(a_{o}\right)-f\left(\left(1-\lambda \right)|a_{o}-b_{o}|\right))+{\left(1-\lambda \right)}^{\alpha }(f\left(b_{o}\right)-f\left(\lambda |a_{o}-b_{o}|\right))), \end{array}$$

*and*

$$\begin{array}{c} g(\lambda a_{o}+\left(1-\lambda \right)b_{o})\leq \lambda^{\alpha }(g\left(a_{o}\right)-g\left(\left(1-\lambda \right)|a_{o}-b_{o}|\right))+{\left(1-\lambda \right)}^{\alpha }(g\left(b_{o}\right)-g\left(\lambda |a_{o}-b_{o}|\right))), \end{array}$$

*now*

$$\begin{array}{cc} & \left(f+g\right)(\lambda a_{o}+\left(1-\lambda \right)b_{o})=f(\lambda a_{o}+\left(1-\lambda \right)b_{o})+g(\lambda a_{o}+\left(1-\lambda \right)b_{o})\\ & \leq \lambda^{\alpha }(f\left(a_{o}\right)-f\left(\left(1-\lambda \right)|a_{o}-b_{o}|\right))+{\left(1-\lambda \right)}^{\alpha }(f\left(b_{o}\right)-f\left(\lambda |a_{o}-b_{o}|\right)))\\ & +\lambda^{\alpha }(g\left(a_{o}\right)-g\left(\left(1-\lambda \right)|a_{o}-b_{o}|\right))+{\left(1-\lambda \right)}^{\alpha }(g\left(b_{o}\right)-g\left(\lambda |a_{o}-b_{o}|\right)))\\ & =\lambda^{\alpha }(\left(f+g\right)\left(a_{o}\right)-\left(f+g\right)\left(\left(1-\lambda \right)|a_{o}-b_{o}|\right))+{\left(1-\lambda \right)}^{\alpha }(\left(f+g\right)\left(b_{o}\right)-\left(f+g\right)\left(\lambda |a_{o}-b_{o}|\right))). \end{array}$$

*Thus*

$$\begin{array}{cc} & \left(f+g\right)(\lambda a_{o}+\left(1-\lambda \right)b_{o})\leq \lambda^{\alpha }(\left(f+g\right)\left(a_{o}\right)-\left(f+g\right)\left(\left(1-\lambda \right)|a_{o}-b_{o}|\right))\\ & +{\left(1-\lambda \right)}^{\alpha }(\left(f+g\right)\left(b_{o}\right)-\left(f+g\right)\left(\lambda |a_{o}-b_{o}|\right))), \end{array}$$ (4)

*similarly*

$$\begin{array}{cc} & \text{K}f(\lambda a_{o}+\left(1-\lambda \right)b_{o})\leq \text{K}((\lambda^{\alpha }(f\left(a_{o}\right)-f\left(\left(1-\lambda \right)|a_{o}-b_{o}|\right))+{\left(1-\lambda \right)}^{\alpha }(f\left(b_{o}\right)-f\left(\lambda |a_{o}-b_{o}|\right))))\\ & =(\lambda^{\alpha }(\text{K}f\left(a_{o}\right)-\text{K}f\left(\left(1-\lambda \right)|a_{o}-b_{o}|\right))+{\left(1-\lambda \right)}^{\alpha }(\text{K}f\left(b_{o}\right)-\text{K}f\left(\lambda |a_{o}-b_{o}|\right))), \end{array}$$

*thus*

$$\begin{array}{cc} & \text{K}f(\lambda a_{o}+\left(1-\lambda \right)b_{o})\leq (\lambda^{\alpha }(\text{K}f\left(a_{o}\right)-\text{K}f\left(\left(1-\lambda \right)|a_{o}-b_{o}|\right))\\ & +{\left(1-\lambda \right)}^{\alpha }(\text{K}f\left(b_{o}\right)-\text{K}f\left(\lambda |a_{o}-b_{o}|\right))). \end{array}$$ (5)

*Hence from* (4) *and* (5) *we conclude that* f+g *and* Kf, *are generalized superquadratic functions*

**Proposition 2** *If* f *is a generalised superquadratic function and* g *is a convex function on I, then* f∘g *is also a generalised superquadratic function*.

***proof***: *Let* $a_{o},b_{o}\in I,\lambda \in \left(0,1\right)$ *and* 0 < *α* ≤ 1. *Based on the stated assumptions, we are left with*

$$\begin{array}{cc} & \left(f\circ g\right)(\lambda a_{o}+\left(1-\lambda \right)b_{o})\\ & =f(g(\lambda a_{o}+\left(1-\lambda \right)b_{o}))\\ & \leq f(\lambda g(a_{o})+\left(1-\lambda \right)g(b_{o}))\\ & \leq \lambda^{\alpha }(\text{f}\left(g\left(a_{o}\right)\right)-\text{f}\left(\left(1-\lambda \right)|g\left(a_{o}\right)-g\left(b_{o}\right)|\right))+{\left(1-\lambda \right)}^{\alpha }(\text{f}\left(g\left(b_{o}\right)\right)-\text{f}\left(\lambda |g\left(a_{o}\right)-g\left(b_{o}\right)|\right)), \end{array}$$

*this demonstrates that* f∘g *is a generalised superquadratic function*.

For an assessment of the validity of Proposition 2, take a look at the example below.

**Fig 7:**
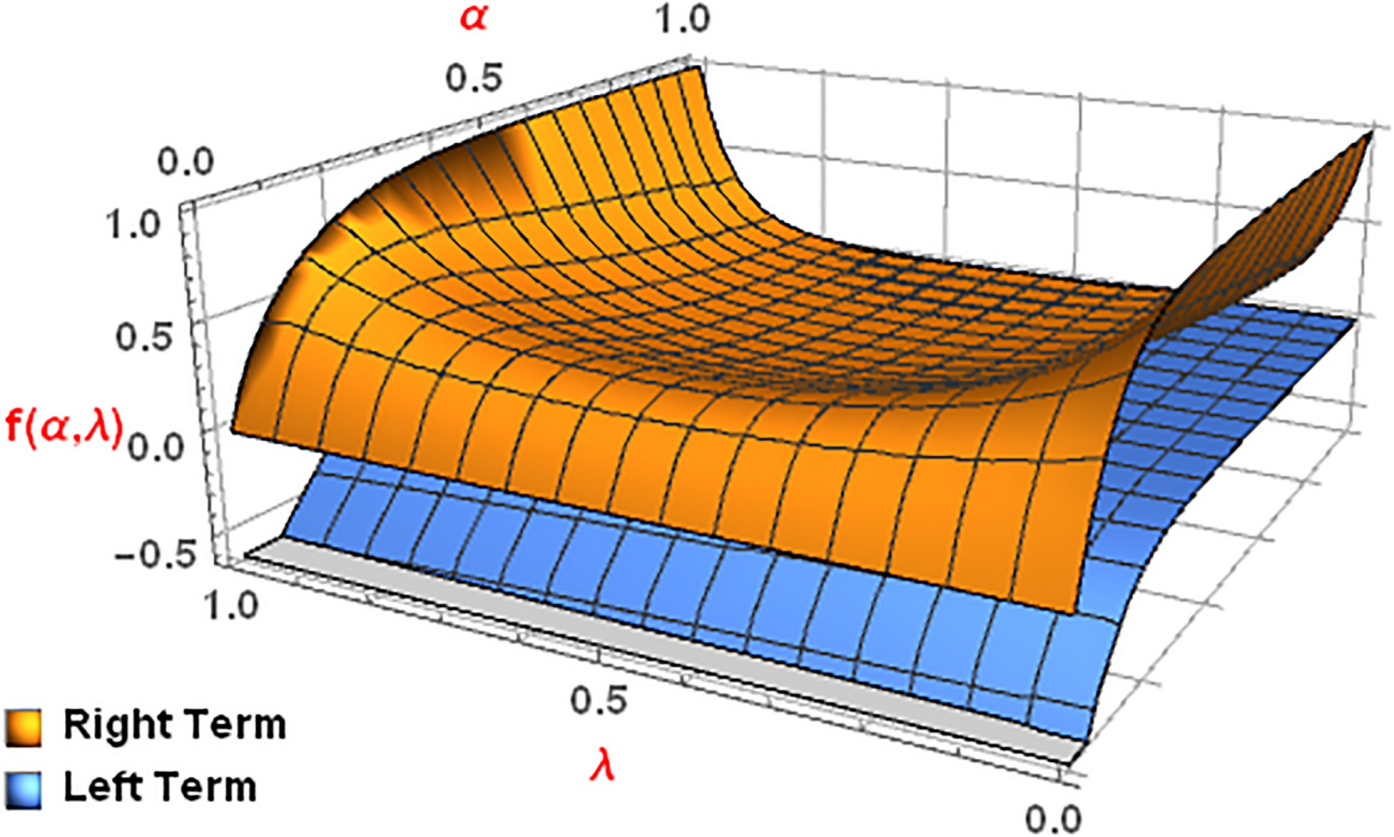
Graphical description of (f∘g)(ϰ).

**Example 4** *Since we know that* $f\left(\varkappa \right)=-{\left(\varkappa +\text{k}\right)}^{-\alpha p}$ *is a generalized superquadratic function while* $f\left(\varkappa \right)=\varkappa^{2}$ *is a convex funtion on [3, 6] then* $f\circ g\left(\varkappa \right)=-{\left(\varkappa +\text{k}\right)}^{-2\alpha p}$ *satisfying Definition 4 for* $p=3,a_{o}=3,b_{o}=6,\text{k}=1,\;\;0<\alpha \leq 1$ *and* (0 < λ ≤ 1). *The following* Fig 7 *of* (f∘g)(ϰ) *confirms the statement of Proposition 2*.

**Theorem 3** *Let* f *is a generalized superquadratic function then*

*(i)* $f\left(0\right)\leq 0^{\alpha }$ ,
*(ii) If* $f\left(\varkappa \right)\geq 0^{\alpha },\;\;\;\varkappa^{\alpha }\geq 0^{\alpha }$ *then* f *is a generalized convex and* $f\left(0\right)=0^{\alpha }$ .

*(i)* ***proof***: *Considering the definition of generalised superquadratic functions*.

$$\begin{array}{c} f(\lambda \varkappa +\left(1-\lambda \right){\text{y}}_{o})\leq \lambda^{\alpha }(f\left(\varkappa \right)-f(\left(1-\lambda \right)|\varkappa -{\text{y}}_{o}|))+{\left(1-\lambda \right)}^{\alpha }(f\left({\text{y}}_{o}\right)-f\left(\lambda |\varkappa -{\text{y}}_{o}|\right))), \end{array}$$ (6)

*setting ϰ* = y_*o*_ *in* (6) *we get*

$$\begin{array}{cc} & f(\varkappa )\leq \lambda^{\alpha }(f(\varkappa )-f(0))+{\left(1-\lambda \right)}^{\alpha }(f(\varkappa )-f(0)))\\ & =\lambda^{\alpha }(f(\varkappa )-f(0))+\left(1-\lambda^{\alpha }\right)(f(\varkappa )-f(0)))\\ & =f(\varkappa )-f(0), \end{array}$$

*it implies that*

$$\begin{array}{c} f\left(0\right)\leq 0^{\alpha }. \end{array}$$ (7)

*(ii)* ***proof***: *Let* f *is a non-negative generalized superquadratic function then*

$$\begin{array}{cc} & f(\lambda \varkappa +\left(1-\lambda \right){\text{y}}_{o})\leq \lambda^{\alpha }(f\left(\varkappa \right)-f(\left(1-\lambda \right)|\varkappa -{\text{y}}_{o}|))+{\left(1-\lambda \right)}^{\alpha }(f\left({\text{y}}_{o}\right)-f\left(\lambda |\varkappa -{\text{y}}_{o}|\right)))\\ & \leq \lambda^{\alpha }f\left(\varkappa \right)+{\left(1-\lambda \right)}^{\alpha }f\left({\text{y}}_{o}\right). \end{array}$$

*This suggests that* f *is a generalised convex function. To prove the 2nd part, we go over* (7).

$$\begin{array}{c} f\left(0\right)\leq 0^{\alpha }, \end{array}$$ (8)

*it is given that* f *is a non-negative. It implies that*

$$\begin{array}{c} f\left(0\right)\geq 0^{\alpha }, \end{array}$$ (9)

*from* (8) *and* (9) *we have*

$$\begin{array}{c} f\left(0\right)=0^{\alpha }. \end{array}$$

*This overs the proof*.

**Theorem 4** *Let* $f:I\subset \text{R}\to {\text{R}}^{\alpha }$ *be a function for all points* *ϰ*_1_, *ϰ*_2_, *ϰ*_3_ ∈ *I*, *such that ϰ*_1_ < *ϰ*_2_ < *ϰ*_3_. *If* f *is a generalized superquadratic function then the inequality*

$$\begin{array}{c} \begin{array}{c} \left|\begin{array}{ccc} {\varkappa_{1}}^{\alpha } & f(\varkappa_{2}-\varkappa_{1}) & 1^{\alpha }\\ {\varkappa_{2}}^{\alpha } & 0^{\alpha } & 1^{\alpha }\\ {\varkappa_{3}}^{\alpha } & f(\varkappa_{3}-\varkappa_{2}) & 1^{\alpha } \end{array}\right|\leq |\begin{array}{ccc} {\varkappa_{1}}^{\alpha } & f(\varkappa_{1}) & 1^{\alpha }\\ {\varkappa_{2}}^{\alpha } & f(\varkappa_{2}) & 1^{\alpha }\\ {\varkappa_{3}}^{\alpha } & f(\varkappa_{3}) & 1^{\alpha } \end{array}| \end{array} \end{array}$$ (10)

*holds*.

***proof***: *Let* f *is a generalized superquadratic function. Then*

$$\begin{array}{cc} & f(\lambda \varkappa +\left(1-\lambda \right){\text{y}}_{o})\leq \lambda^{\alpha }(f\left(\varkappa \right)-f(\left(1-\lambda \right)|\varkappa -{\text{y}}_{o}|))+{\left(1-\lambda \right)}^{\alpha }(f\left({\text{y}}_{o}\right)-f\left(\lambda |\varkappa -{\text{y}}_{o}|\right))), \end{array}$$ (11)

*setting*

$$\begin{array}{c} \varkappa_{2}=\lambda \varkappa +\left(1-\lambda \right){\text{y}}_{o}. \end{array}$$ (12)

*Putting* *ϰ* = *ϰ*_1_ *and* y_*o*_ = *ϰ*_3_ *in* (12) *we get*

$$\begin{array}{cc} & \varkappa_{2}=\lambda \varkappa_{1}+\left(1-\lambda \right)\varkappa_{3}.\\ & \Rightarrow \lambda =\frac{\varkappa_{3}-\varkappa_{2}}{\varkappa_{3}-\varkappa_{1}}. \end{array}$$

*Next substituting* *ϰ* = *ϰ*_1_, y_*o*_ = *ϰ*_3_ *and* $\lambda =\frac{\varkappa_{3}-\varkappa_{2}}{\varkappa_{3}-\varkappa_{1}}$ *in* (11) *we get*

$$\begin{array}{cc} & f\left(\varkappa_{2}\right)\leq {\left(\frac{\varkappa_{3}-\varkappa_{2}}{\varkappa_{3}-\varkappa_{1}}\right)}^{\alpha }\left(f\left(\varkappa_{1}\right)-f\left(|\varkappa_{2}-\varkappa_{1}|\right)\right)+{\left(\frac{\varkappa_{2}-\varkappa_{1}}{\varkappa_{3}-\varkappa_{1}}\right)}^{\alpha }\left(f\left(\varkappa_{3}\right)-f\left(\varkappa_{3}-\varkappa_{2}\right)\right), \end{array}$$

*or*

$$\begin{array}{cc} & {\left(\varkappa_{3}-\varkappa_{1}\right)}^{\alpha }f\left(\varkappa_{2}\right)\leq {\left(\varkappa_{3}-\varkappa_{2}\right)}^{\alpha }\left(f\left(\varkappa_{1}\right)-f\left(|\varkappa_{2}-\varkappa_{1}|\right)\right)+{\left(\varkappa_{2}-\varkappa_{1}\right)}^{\alpha }\left(f\left(\varkappa_{3}\right)-f\left(\varkappa_{3}-\varkappa_{2}\right)\right).\\ & \Rightarrow {\left(\varkappa_{3}-\varkappa_{2}\right)}^{\alpha }\left(f\left(\varkappa_{1}\right)-f\left(|\varkappa_{2}-\varkappa_{1}|\right)\right)+{\left(\varkappa_{2}-\varkappa_{1}\right)}^{\alpha }\left(f\left(\varkappa_{3}\right)-f\left(\varkappa_{3}-\varkappa_{2}\right)\right)\\ & -{\left(\varkappa_{3}-\varkappa_{1}\right)}^{\alpha }f\left(\varkappa_{2}\right)\geq 0. \end{array}$$

*Hence we have*

$$\begin{array}{c} \begin{array}{cc} \left|\begin{array}{ccc} {\varkappa_{1}}^{\alpha } & f(\varkappa_{1}) & 1^{\alpha }\\ {\varkappa_{2}}^{\alpha } & f(\varkappa_{2}) & 1^{\alpha }\\ {\varkappa_{3}}^{\alpha } & f(\varkappa_{3}) & 1^{\alpha } \end{array}\right|-|\begin{array}{ccc} {\varkappa_{1}}^{\alpha } & f(\varkappa_{2}-\varkappa_{1}) & 1^{\alpha }\\ {\varkappa_{2}}^{\alpha } & 0^{\alpha } & 1^{\alpha }\\ {\varkappa_{3}}^{\alpha } & f(\varkappa_{3}-\varkappa_{2}) & 1^{\alpha } \end{array}| & \geq 0. \end{array} \end{array}$$

We consider the following example to know that how the statement (10) works for conforming the definition of generalized superquadraticity of a function.

**Example 5** *Let we have a generalised superquadratic function*.

$$\begin{array}{c} E_{\alpha }\left(\varkappa^{\alpha }\right)=\sum_{k=0}^{\infty }\frac{\varkappa^{k\alpha }}{\Gamma (1+k\alpha )},\;\varkappa \in R, \end{array}$$

*for all ϰ* ≥ 3, *where* (0 < *α* ≤ 1).

*Suppose that ϰ*_1_ = 3, *ϰ*_2_ = 4 *and ϰ*_3_ = 5 *then we have*

$$\begin{array}{c} \begin{array}{c} \left|\begin{array}{ccc} {\varkappa_{1}}^{\alpha } & f(\varkappa_{1}) & 1^{\alpha }\\ {\varkappa_{2}}^{\alpha } & f(\varkappa_{2}) & 1^{\alpha }\\ {\varkappa_{3}}^{\alpha } & f(\varkappa_{3}) & 1^{\alpha } \end{array}\right|=E_{\alpha }\left(3^{\alpha }\right)+E_{\alpha }\left(5^{\alpha }\right)-2^{\alpha }E_{\alpha }\left(4^{\alpha }\right) \end{array} \end{array}$$

*and*

$$\begin{array}{c} \begin{array}{c} \left|\begin{array}{ccc} {\varkappa_{1}}^{\alpha } & f(\varkappa_{2}-\varkappa_{1}) & 1^{\alpha }\\ {\varkappa_{2}}^{\alpha } & 0^{\alpha } & 1^{\alpha }\\ {\varkappa_{3}}^{\alpha } & f(\varkappa_{3}-\varkappa_{2}) & 1^{\alpha } \end{array}\right|=2^{\alpha }E_{\alpha }\left(1^{\alpha }\right) \end{array} \end{array}$$

**Fig 8:**
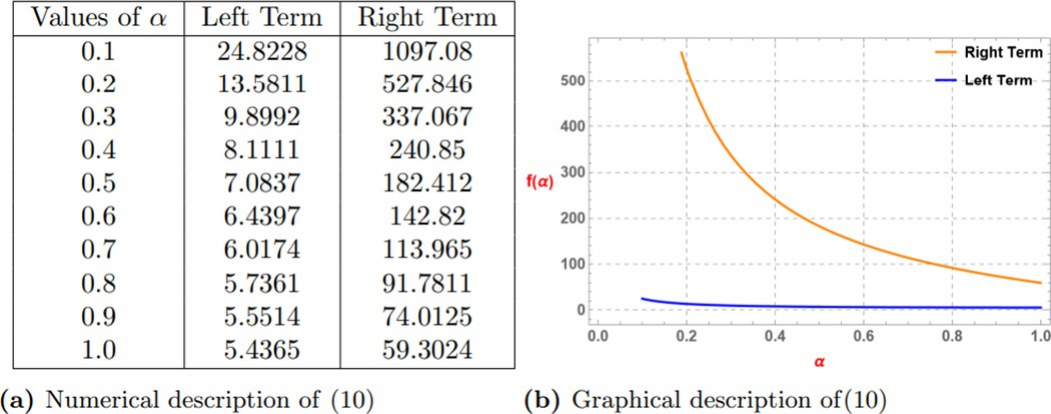
Numerical and graphical description of (10) for (0 < *α* ≤ 1). (**a**) Numerical description of (10). (**b**) Graphical description of (10).

*The subsequent Table 8a and Graph 8b given by* Fig 8 *confirm the validity of the statement of Theorem 4*.

*In order to see its 3D depiction we assume that* $\varkappa_{1}=a_{o},\varkappa_{2}=\frac{a_{o}+b_{o}}{2}$ *and* $\varkappa_{3}=b_{o}$ *for the same function. Then using these values in* (10), *we attain the undermentioned values for right and left terms*.

$$\begin{array}{cc} & Left\;\;\;\;\;Term={\left(b_{o}-a_{o}\right)}^{\alpha }E_{\alpha }({\left(\frac{b_{o}-a_{o}}{2}\right)}^{\alpha })\\ & Right\;\;\;\;\;Term=(\frac{b_{o}-a_{o}}{2}{)}^{\alpha }\left[E_{\alpha }\left({b_{o}}^{\alpha }\right)+E_{\alpha }\left({a_{o}}^{\alpha }\right)\right]-{\left(b_{o}-a_{o}\right)}^{\alpha }E_{\alpha }({\left(\frac{a_{o}+b_{o}}{2}\right)}^{\alpha }). \end{array}$$

**Fig 9:**
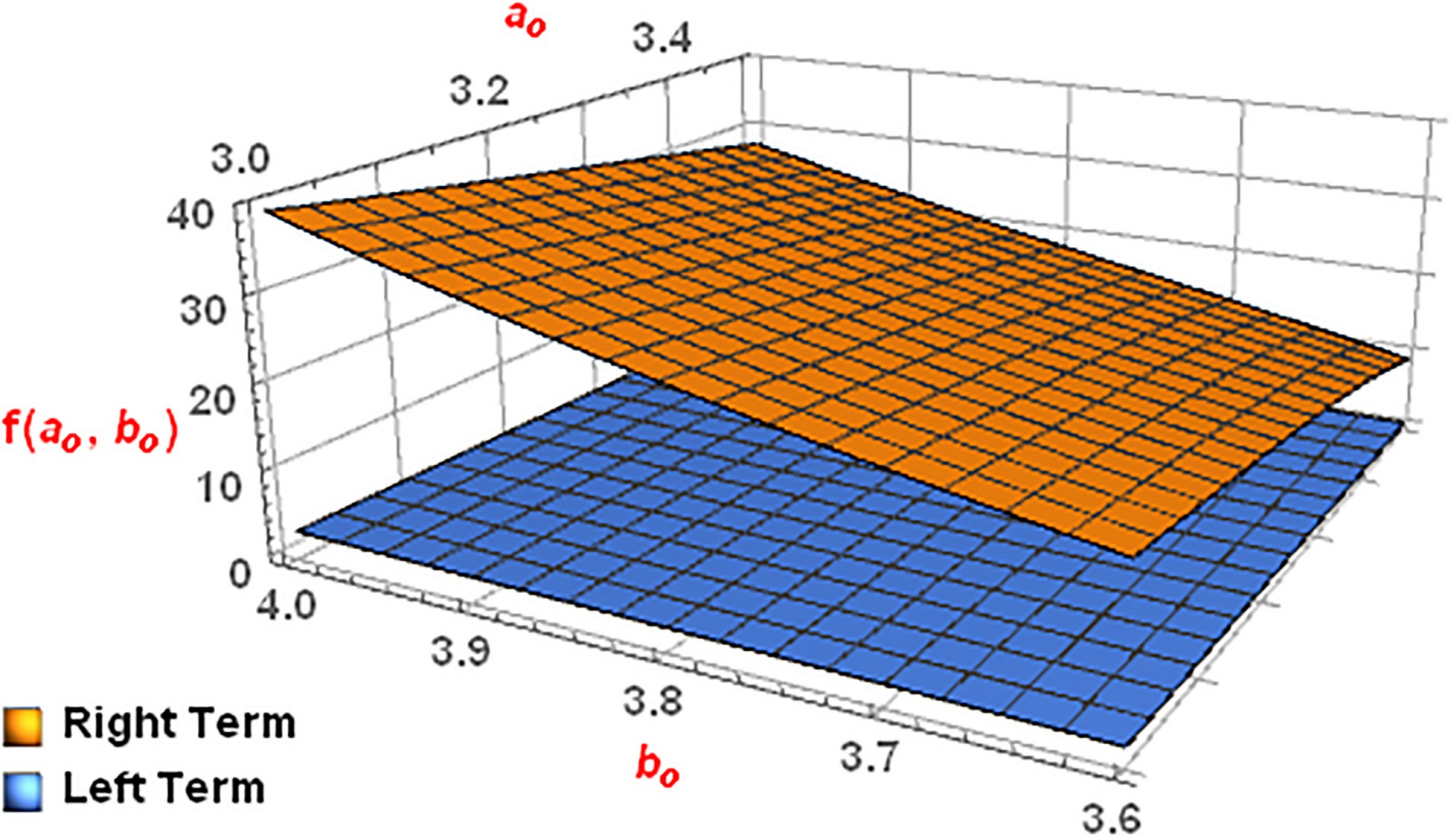
Graphical description for $\alpha =\frac{1}{2}$, $a_{o}\in \left[3,3.5\right]$ and $b_{o}\in \left[3.6,4\right]$.

*The* Fig 9 *for the aforementioned Left and Right terms confirms the validity of the statement of Theorem 4*.

**Example 6** *Let we have a generalised superquadratic function*

$$\begin{array}{c} \text{f}\left(\varkappa \right)=-{\left(\varkappa +\text{k}\right)}^{-\alpha p},\forall \;\;\;\text{k}>0, \end{array}$$

*for all ϰ* ≥ 0 *and* p≥1, *where* (0 < *α* ≤ 1).

*Suppose that* $\text{k}=1,p=2,\varkappa_{1}=3,\varkappa_{2}=4$ *and* $\varkappa_{3}=5$ *then we have*

$$\begin{array}{c} \begin{array}{c} \left|\begin{array}{ccc} {\varkappa_{1}}^{\alpha } & f\left(\varkappa_{1}\right) & 1^{\alpha }\\ {\varkappa_{2}}^{\alpha } & f\left(\varkappa_{2}\right) & 1^{\alpha }\\ {\varkappa_{3}}^{\alpha } & f\left(\varkappa_{3}\right) & 1^{\alpha } \end{array}\right|={\left(\frac{4}{45}\right)}^{\alpha }-{\left(\frac{11}{300}\right)}^{\alpha }-{\left(\frac{1}{16}\right)}^{\alpha } \end{array} \end{array}$$

*and*

$$\begin{array}{c} \begin{array}{c} \left|\begin{array}{ccc} {\varkappa_{1}}^{\alpha } & f\left(\varkappa_{2}-\varkappa_{1}\right) & 1^{\alpha }\\ {\varkappa_{2}}^{\alpha } & 0^{\alpha } & 1^{\alpha }\\ {\varkappa_{3}}^{\alpha } & f\left(\varkappa_{3}-\varkappa_{2}\right) & 1^{\alpha } \end{array}\right|={\left(\frac{3}{4}\right)}^{\alpha }-{\left(\frac{1}{4}\right)}^{\alpha }-{\left(1\right)}^{\alpha } \end{array} \end{array}$$

**Fig 10:**
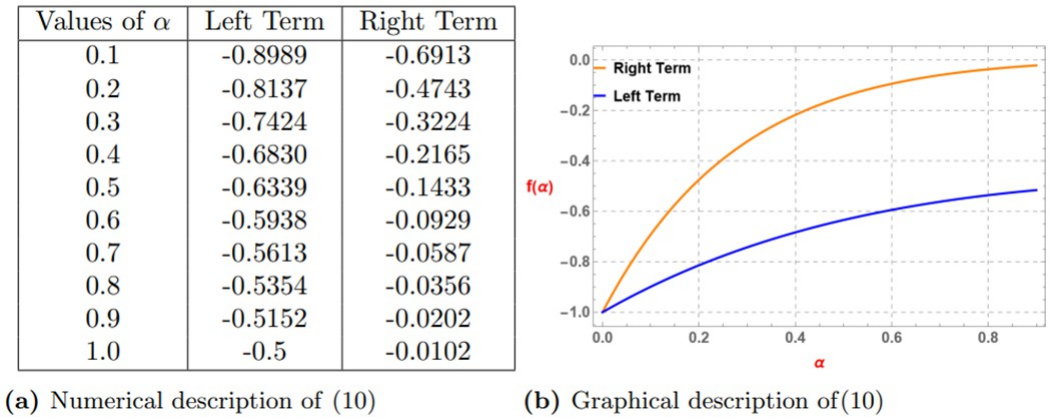
Numerical and graphical description of $\text{f}\left(\varkappa \right)=-{\left(\varkappa +\text{k}\right)}^{-\alpha p}$ satisfying (10) for $\text{k}=1,p=2,\varkappa_{1}=3,\varkappa_{2}=4$ and $\varkappa_{3}=5$ and (0 < *α* ≤ 1). (**a**) Numerical description of (10). (**b**) Graphical description of (10).

*The subsequent Table 10a and Graph 10b given by* Fig 10 *confirm the validity of the statement of Theorem 6*.

**Theorem 5 *(Generalized Jensen inequality)*** Let *ϰ*_*i*_, λ_*i*_ ≥ 0. *i* = 1, …, *n*, $\text{f}:\text{I}\to R^{\alpha }$. If f is a generalized superquadratic function on **I** then

$$\begin{array}{cc} & \text{f}(\Sigma_{i=1}^{n}\lambda_{i}\varkappa_{i})\leq \Sigma_{i=1}^{n}\lambda_{i}^{\alpha }\text{f}(\varkappa_{i})-\Sigma_{i=1}^{n}\lambda_{i}^{\alpha }\text{f}(|\varkappa_{i}-\Sigma_{i=1}^{n}\lambda_{i}\varkappa_{i}|), \end{array}$$ (13)

where $\Sigma_{i=1}^{n}\lambda_{i}=1$ and 0 < *α* ≤ 1.

**proof**: Assume that f is superquadratic function. If n=2, then we have

$$\begin{array}{c} \text{f}(\lambda_{1}\varkappa_{1}+\lambda_{2}\varkappa_{2})\leq \lambda_{1}^{\alpha }\text{f}\left(\varkappa_{1}\right)+\lambda_{2}^{\alpha }\text{f}\left(\varkappa_{2}\right)-\lambda_{1}^{\alpha }\text{f}(\lambda_{2}|\varkappa_{1}-\varkappa_{2}\left|\right)-\lambda_{2}^{\alpha }\text{f}(\lambda_{1}\left|\varkappa_{1}-\varkappa_{2}\right|), \end{array}$$

suppose that (13) is valid for n-1, then we have

$$\begin{array}{cc} & \text{f}(\Sigma_{i=1}^{n-1}\lambda_{i}\varkappa_{i})\leq \Sigma_{i=1}^{n-1}\lambda_{i}^{\alpha }\text{f}\left(\varkappa_{i}\right)-\Sigma_{i=1}^{n-1}\lambda_{i}^{\alpha }\text{f}(|\varkappa_{i}-\Sigma_{i=1}^{n-1}\lambda_{i}\varkappa_{i}|), \end{array}$$ (14)

now let us prove that (13) is valid for n.

$$\begin{array}{cc} & \text{f}(\Sigma_{i=1}^{n}\lambda_{i}\varkappa_{i})=\text{f}(\lambda_{n}\varkappa_{n}+\Sigma_{i=1}^{n-1}\lambda_{i}\varkappa_{i})=\text{f}(\lambda_{n}\varkappa_{n}+(\frac{\lambda }{\lambda })\Sigma_{i=1}^{n-1}\lambda_{i}\varkappa_{i}). \end{array}$$

Where $\lambda =\Sigma_{i=1}^{n-1}\lambda_{i}$

$$\begin{array}{cc} & \text{f}\left(\Sigma_{i=1}^{n}\lambda_{i}\varkappa_{i}\right)\leq \lambda_{n}^{\alpha }\text{f}\left(\varkappa_{n}\right)+\lambda^{\alpha }\text{f}(\Sigma_{i=1}^{n-1}(\frac{\lambda_{i}\varkappa_{i}}{\lambda }))\\ & -\lambda_{n}^{\alpha }\text{f}(\lambda |\varkappa_{n}-\Sigma_{i=1}^{n-1}(\frac{\lambda_{i}\varkappa_{i}}{\lambda })|)-\lambda^{\alpha }\text{f}(\lambda_{n}|\varkappa_{n}-\Sigma_{i=1}^{n-1}(\frac{\lambda_{i}\varkappa_{i}}{\lambda })|). \end{array}$$ (15)

Using (14) in (15) then we obtain

$$\begin{array}{cc} & \text{f}\left(\Sigma_{i=1}^{n}\lambda_{i}\varkappa_{i}\right)\leq \lambda_{n}^{\alpha }\text{f}\left(\varkappa_{n}\right)+\Sigma_{i=1}^{n-1}\lambda_{i}^{\alpha }\text{f}\left(\varkappa_{i}\right)-\Sigma_{i=1}^{n-1}\lambda_{i}^{\alpha }\text{f}(|\varkappa_{i}-\Sigma_{i=1}^{n-1}\lambda_{i}\varkappa_{i}\left|\right)\\ & -\lambda_{n}^{\alpha }\text{f}(\lambda |\varkappa_{n}-\Sigma_{i=1}^{n-1}(\frac{\lambda_{i}\varkappa_{i}}{\lambda })|)-\lambda^{\alpha }\text{f}(\lambda_{n}|\varkappa_{n}-\Sigma_{i=1}^{n-1}(\frac{\lambda_{i}\varkappa_{i}}{\lambda })|)\\ & =\lambda_{n}^{\alpha }\text{f}\left(\varkappa_{n}\right)+\Sigma_{i=1}^{n-1}\lambda_{i}^{\alpha }\text{f}\left(\varkappa_{i}\right)-\Sigma_{i=1}^{n-1}\lambda_{i}^{\alpha }\text{f}(|\varkappa_{i}-\Sigma_{i=1}^{n-1}\lambda_{i}\varkappa_{i}\left|\right)\\ & -\lambda_{n}^{\alpha }\text{f}(|\lambda \varkappa_{n}-\Sigma_{i=1}^{n-1}\lambda_{i}\varkappa_{i})|)-\lambda^{\alpha }\text{f}(\lambda_{n}|\varkappa_{n}-\Sigma_{i=1}^{n-1}(\frac{\lambda_{i}\varkappa_{i}}{\lambda })|)\\ & =\Sigma_{i=1}^{n}\lambda_{i}^{\alpha }\text{f}\left(\varkappa_{i}\right)-\Sigma_{i=1}^{n-1}\lambda_{i}^{\alpha }\text{f}(|\varkappa_{i}-\Sigma_{i=1}^{n-1}\lambda_{i}\varkappa_{i}\left|\right)\\ & -\lambda_{n}^{\alpha }\text{f}(|\left(1-\lambda_{n}\right)\varkappa_{n}-\Sigma_{i=1}^{n-1}\lambda_{i}\varkappa_{i})|)-\lambda^{\alpha }\text{f}(\lambda_{n}|\varkappa_{n}-\Sigma_{i=1}^{n-1}(\frac{\lambda_{i}\varkappa_{i}}{\lambda })|)\\ & =\Sigma_{i=1}^{n}\lambda_{i}^{\alpha }\text{f}\left(\varkappa_{i}\right)-\Sigma_{i=1}^{n-1}\lambda_{i}^{\alpha }\text{f}(|\varkappa_{i}-\Sigma_{i=1}^{n-1}\lambda_{i}\varkappa_{i}\left|\right)\\ & -\lambda_{n}^{\alpha }\text{f}(|\varkappa_{n}-\lambda_{n}\varkappa_{n}-\Sigma_{i=1}^{n-1}\lambda_{i}\varkappa_{i})|)-\lambda^{\alpha }\text{f}(\lambda_{n}|\varkappa_{n}-\Sigma_{i=1}^{n-1}(\frac{\lambda_{i}\varkappa_{i}}{\lambda })|). \end{array}$$

It implies that

$$\begin{array}{cc} & \text{f}\left(\Sigma_{i=1}^{n}\lambda_{i}\varkappa_{i}\right)\leq \Sigma_{i=1}^{n}\lambda_{i}^{\alpha }\text{f}\left(\varkappa_{i}\right)-\Sigma_{i=1}^{n-1}\lambda_{i}^{\alpha }\text{f}(|\varkappa_{i}-\Sigma_{i=1}^{n-1}\lambda_{i}\varkappa_{i}\left|\right)\\ & -\lambda_{n}^{\alpha }\text{f}(|\varkappa_{n}-\Sigma_{i=1}^{n}\lambda_{i}\varkappa_{i})|)\\ & =\Sigma_{i=1}^{n}\lambda_{i}^{\alpha }\text{f}\left(\varkappa_{i}\right)-\Sigma_{i=1}^{n}\lambda_{i}^{\alpha }\text{f}(|\varkappa_{i}-\Sigma_{i=1}^{n}\lambda_{i}\varkappa_{i}\left|\right). \end{array}$$

Hence the proof.

**Remark 2** *If α = 1 is taken in Theorem 5 then we get the Jensen iequality for superquadratic function*.

**Theorem 6 *(Generalized converse Jensen inequality)*** Let $f:I\subset \text{R}\to {\text{R}}^{\alpha }$ be a generalized superquadratic function. Let (m_*o*_, M_*o*_) ⊆ I then for *ϰ*_1_, *ϰ*_2_, …, *ϰ*_*n*_ ∈ (m_*o*_, M_*o*_) we have

$$\begin{array}{cc} & \sum_{i=1}^{n}\lambda_{i}^{\alpha }f(\varkappa_{i})\leq \sum_{i=1}^{n}\lambda_{i}^{\alpha }[(\frac{{\text{M}}_{o}^{\alpha }-\varkappa_{i}^{\alpha }}{{\text{M}}_{o}^{\alpha }-{\text{m}}_{o}^{\alpha }})f\left({\text{m}}_{o}\right)+(\frac{\varkappa_{i}^{\alpha }-{\text{m}}_{o}^{\alpha }}{{\text{M}}_{o}^{\alpha }-{\text{m}}_{o}^{\alpha }})f\left({\text{M}}_{o}\right)]\\ & -\sum_{i=1}^{n}\lambda_{i}^{\alpha }[(\frac{{\text{M}}_{o}^{\alpha }-\varkappa_{i}^{\alpha }}{{\text{M}}_{o}^{\alpha }-{\text{m}}_{o}^{\alpha }})f\left(\varkappa_{i}-{\text{m}}_{o}\right)+(\frac{\varkappa_{i}^{\alpha }-{\text{m}}_{o}^{\alpha }}{{\text{M}}_{o}^{\alpha }-{\text{m}}_{o}^{\alpha }})f\left({\text{M}}_{o}-\varkappa_{i}\right)]. \end{array}$$ (16)

**proof**: It can be proved by substituting *ϰ*_1_ = m_*o*_, *ϰ*_2_ = *ϰ*_*i*_ and *ϰ*_3_ = M_*o*_ in (10), such that

$$\begin{array}{c} \begin{array}{c} \left|\begin{array}{ccc} {\text{m}}_{o}^{\alpha } & f({\text{m}}_{o}) & 1^{\alpha }\\ {\varkappa_{i}}^{\alpha } & f(\varkappa_{i}) & 1^{\alpha }\\ {\text{M}}_{o}^{\alpha } & f({\text{M}}_{o}) & 1^{\alpha } \end{array}\right|\geq |\begin{array}{ccc} {\text{m}}_{o}^{\alpha } & f(\varkappa_{i}-{\text{m}}_{o}) & 1^{\alpha }\\ {\varkappa_{i}}^{\alpha } & 0^{\alpha } & 1^{\alpha }\\ {\text{M}}_{o}^{\alpha } & f({\text{M}}_{o}-\varkappa_{i}) & 1^{\alpha } \end{array}|. \end{array} \end{array}$$

It implies that

$$\begin{array}{cc} & f(\varkappa_{i})\leq (\frac{{\text{M}}_{o}^{\alpha }-\varkappa_{i}^{\alpha }}{{\text{M}}_{o}^{\alpha }-{\text{m}}_{o}^{\alpha }})f\left({\text{m}}_{o}\right)+(\frac{\varkappa_{i}^{\alpha }-{\text{m}}_{o}^{\alpha }}{{\text{M}}_{o}^{\alpha }-{\text{m}}_{o}^{\alpha }})f\left({\text{M}}_{o}\right)\\ & -(\frac{{\text{M}}_{o}^{\alpha }-\varkappa_{i}^{\alpha }}{{\text{M}}_{o}^{\alpha }-{\text{m}}_{o}^{\alpha }})f\left(\varkappa_{i}-{\text{m}}_{o}\right)+(\frac{\varkappa_{i}^{\alpha }-{\text{m}}_{o}^{\alpha }}{{\text{M}}_{o}^{\alpha }-{\text{m}}_{o}^{\alpha }})f\left({\text{M}}_{o}-\varkappa_{i}\right). \end{array}$$

Multiplying both sides by $\lambda_{i}^{\alpha }$ and then summing up to *n* we get the required result.

The subsequent result discusses the Mercer-Jensen’s type inequality for generalised superquadratic functions.

**Theorem 7 *(Generalized Mercer-Jensen’s inequality)*** Let $f\in I_{\varkappa }^{\left(\alpha \right)}\left[a_{o},b_{o}\right]$ be a generalized superquadratic function defined on a real interval $I=[a_{o},b_{o}]$ and finite positive increasing sequence ${\left\{\varkappa_{\text{k}}\right\}}_{\text{k}=1}^{\text{n}}\in I$ where 1 ≤ k ≤ n, we have

$$\begin{array}{cc} & f(\varkappa_{1}+\varkappa_{\text{n}}-\sum_{\text{k}=1}^{\text{n}}\lambda_{\text{k}}\varkappa_{\text{k}})\\ \leq & f\left(\varkappa_{1}\right)+f\left(\varkappa_{n}\right)-\sum_{\text{k}=1}^{\text{n}}\lambda_{\text{k}}^{\alpha }[f\left(\varkappa_{\text{k}}-\varkappa_{1}\right)+f\left(\varkappa_{n}-\varkappa_{\text{k}}\right)]\\ & -\sum_{\text{k}=1}^{\text{n}}\lambda_{\text{k}}^{\alpha }f\left(\varkappa_{\text{k}}\right)-\sum_{\text{k}=1}^{\text{n}}\lambda_{\text{k}}^{\alpha }f(|\varkappa_{\text{k}}-\sum_{\text{j}=1}^{\text{n}}\lambda_{\text{j}}\varkappa_{\text{j}}|), \end{array}$$ (17)

∀ λ_k_ ∈ [0, 1] such that $\sum_{\text{k}=1}^{\text{n}}\lambda_{\text{k}}=1$.

**proof**: Let *ϰ*_k_ = *α*_k_*ϰ*_1_ + *β*_k_*ϰ*_2_ such that *α*_k_ + *β*_k_ = 1, then we have

$$\begin{array}{cc} & f\left(\beta_{\text{k}}\varkappa_{1}+\alpha_{\text{k}}\varkappa_{2}\right)=f\left(\varkappa_{1}+\varkappa_{\text{n}}-\varkappa_{\text{k}}\right)\\ & \leq f\left(\varkappa_{n}\right)+f\left(\varkappa_{1}\right)-f(\alpha_{\text{k}}|\varkappa_{1}-\varkappa_{n}|)-f(\beta_{\text{k}}|\varkappa_{1}-\varkappa_{n}\left|\right)-f\left(\varkappa_{\text{k}}\right), \end{array}$$ (18)

next consider the L.H.S of (17), we get

$$\begin{array}{c} f(\varkappa_{1}+\varkappa_{\text{n}}-\sum_{\text{k}=1}^{\text{n}}\lambda_{\text{k}}\varkappa_{\text{k}})=f(\sum_{\text{k}=1}^{\text{n}}\lambda_{\text{k}}\left(\varkappa_{1}+\varkappa_{\text{n}}-\varkappa_{\text{k}}\right)), \end{array}$$

using the result of Theorem 5, we get

$$\begin{array}{cc} & f(\varkappa_{1}+\varkappa_{\text{n}}-\sum_{\text{k}=1}^{\text{n}}\lambda_{\text{k}}\varkappa_{\text{k}})\leq \sum_{\text{k}=1}^{\text{n}}\lambda_{\text{k}}^{\alpha }f\left(\varkappa_{1}+\varkappa_{\text{n}}-\varkappa_{\text{k}}\right)\\ & -\sum_{\text{k}=1}^{\text{n}}\lambda_{\text{k}}^{\alpha }f(|\left(\varkappa_{1}+\varkappa_{\text{n}}-\varkappa_{\text{k}}\right)-\sum_{\text{j}=1}^{\text{n}}\lambda_{\text{j}}\left(\varkappa_{1}+\varkappa_{\text{n}}-\varkappa_{\text{j}}\right)|), \end{array}$$ (19)

using *ϰ*_k_ = *α*_k_*ϰ*_1_ + *β*_k_*ϰ*_*n*_ in the first term on the R.H.S of (19) and then utilizing the result (18), we get

$$\begin{array}{cc} & f(\varkappa_{1}+\varkappa_{\text{n}}-\sum_{\text{k}=1}^{\text{n}}\lambda_{\text{k}}\varkappa_{\text{k}})\leq \sum_{\text{k}=1}^{\text{n}}\lambda_{\text{k}}^{\alpha }f\left(\alpha_{\text{k}}\varkappa_{n}+\beta_{\text{k}}\varkappa_{1}\right)-\sum_{\text{k}=1}^{\text{n}}\lambda_{\text{k}}^{\alpha }f(|\varkappa_{\text{k}}-\sum_{\text{j}=1}^{\text{n}}\lambda_{\text{j}}\varkappa_{\text{j}}|)\\ & \leq \sum_{\text{k}=1}^{\text{n}}\lambda_{\text{k}}^{\alpha }[f\left(\varkappa_{n}\right)+f\left(\varkappa_{1}\right)-f(\alpha_{\text{k}}|\varkappa_{1}-\varkappa_{n}|)-f(\beta_{\text{k}}|\varkappa_{1}-\varkappa_{n}\left|\right)-f\left(\varkappa_{\text{k}}\right)]\\ & -\sum_{\text{k}=1}^{\text{n}}\lambda_{\text{k}}^{\alpha }f(|\varkappa_{\text{k}}-\sum_{\text{j}=1}^{\text{n}}\lambda_{\text{j}}\varkappa_{\text{j}}|)=f\left(\varkappa_{n}\right)+f\left(\varkappa_{1}\right)-\sum_{\text{k}=1}^{\text{n}}\lambda_{\text{k}}^{\alpha }[f(\alpha_{\text{k}}|\varkappa_{1}-\varkappa_{n}|)+f(\beta_{\text{k}}|\varkappa_{1}-\varkappa_{n}\left|\right)]\\ & -\sum_{\text{k}=1}^{\text{n}}\lambda_{\text{k}}^{\alpha }f\left(\varkappa_{\text{k}}\right)-\sum_{\text{k}=1}^{\text{n}}\lambda_{\text{k}}^{\alpha }f(|\varkappa_{\text{k}}-\sum_{\text{j}=1}^{\text{n}}\lambda_{\text{j}}\varkappa_{\text{j}}|), \end{array}$$ (20)

setting $\alpha_{\text{k}}=\frac{\varkappa_{\text{k}}-\varkappa_{1}}{\varkappa_{\text{n}}-\varkappa_{1}}$ and $\beta_{\text{k}}=\frac{\varkappa_{\text{n}}-\varkappa_{\text{k}}}{\varkappa_{\text{n}}-\varkappa_{1}}$ in (20), we get

$$\begin{array}{cc} & f(\varkappa_{1}+\varkappa_{\text{n}}-\sum_{\text{k}=1}^{\text{n}}\lambda_{\text{k}}\varkappa_{\text{k}})\\ \leq & f\left(\varkappa_{1}\right)+f\left(\varkappa_{n}\right)-\sum_{\text{k}=1}^{\text{n}}\lambda_{\text{k}}^{\alpha }[f\left(\varkappa_{\text{k}}-\varkappa_{1}\right)+f\left(\varkappa_{n}-\varkappa_{\text{k}}\right)]\\ & -\sum_{\text{k}=1}^{\text{n}}\lambda_{\text{k}}^{\alpha }f\left(\varkappa_{\text{k}}\right)-\sum_{\text{k}=1}^{\text{n}}\lambda_{\text{k}}^{\alpha }f(|\varkappa_{\text{k}}-\sum_{\text{j}=1}^{\text{n}}\lambda_{\text{j}}\varkappa_{\text{j}}|). \end{array}$$

Hence the proof.

**Remark 3** *If we put α = 1 in Theorem 7 then we get the Mercer-Jensen’s iequality for superquadratic function*.

**Theorem 8 *(Generalized Hermite Hadamard’s inequality)*** Let $f\in I_{\varkappa }^{\left(\alpha \right)}\left[a_{o},b_{o}\right]$ be a generalized superquadratic function on $\left[a_{o},b_{o}\right]$ with $a_{o}<b_{o}$ then for any λ ≥ 0 the subsequent inequality

f(ao+bo2)+Γ(1+α)(bo-ao)αIaoboαf(|ao+bo2-ϰ|)≤Γ(1+α)(bo-ao)αIaoboαf(ϰ)≤f(bo)+f(ao)2α-1(bo-ao)2α∫aobo[(ϰ-ao)αf(bo-ϰ)+(bo-ϰ)αf(ϰ-ao)](dϰ)α.

**proof**: Since f is a generalized superquadratic function then it must satisfies the following inequality

$$\begin{array}{c} f(\lambda \varkappa +\left(1-\lambda \right){\text{y}}_{o})\leq \lambda^{\alpha }(f\left(\varkappa \right)-f(\left(1-\lambda \right)|\varkappa -{\text{y}}_{o}|))+{\left(1-\lambda \right)}^{\alpha }(f\left({\text{y}}_{o}\right)-f\left(\lambda |\varkappa -{\text{y}}_{o}|\right))). \end{array}$$ (21)

Putting $\lambda =\frac{1}{2}$ in (21) we get

$$\begin{array}{cc} & f(\frac{\varkappa +{\text{y}}_{o}}{2})\leq \frac{1}{2^{\alpha }}f\left(\varkappa \right)+\frac{1}{2^{\alpha }}f\left({\text{y}}_{o}\right)-f(\frac{|\varkappa -{\text{y}}_{o}|}{2}). \end{array}$$ (22)

Replacing *ϰ* by $\lambda a_{o}+\left(1-\lambda \right)b_{o}$ and y_*o*_ by $\lambda b_{o}+\left(1-\lambda \right)a_{o}$ in (22), we get.

$$\begin{array}{cc} & f(\frac{a_{o}+b_{o}}{2})\leq \frac{1}{2^{\alpha }}f\left(\lambda a_{o}+\left(1-\lambda \right)b_{o}\right)+\frac{1}{2^{\alpha }}f\left(\lambda b_{o}+\left(1-\lambda \right)a_{o}\right)\\ & -f(\frac{\left(1-2\lambda \right)|b_{o}-a_{o}|}{2}). \end{array}$$ (23)

Integrating w.r.t λ over [0, 1] we have

$$\begin{array}{cc} & f(\frac{a_{o}+b_{o}}{2})\leq \frac{1}{2^{\alpha }}\int_{0}^{1}f\left(\lambda a_{o}+\left(1-\lambda \right)b_{o}\right){\left(d\lambda \right)}^{\alpha }+\frac{1}{2^{\alpha }}\int_{0}^{1}f\left(\lambda b_{o}+\left(1-\lambda \right)a_{o}\right){\left(d\lambda \right)}^{\alpha }\\ & -\int_{0}^{1}f(\frac{\left(1-2\lambda \right)|b_{o}-a_{o}|}{2}){\left(d\lambda \right)}^{\alpha }. \end{array}$$ (24)

It is to be noted that

$$\begin{array}{c} \int_{0}^{1}f\left(\lambda b_{o}+\left(1-\lambda \right)a_{o}\right){\left(d\lambda \right)}^{\alpha }=\int_{0}^{1}f\left(\lambda a_{o}+\left(1-\lambda \right)b_{o}\right){\left(d\lambda \right)}^{\alpha }. \end{array}$$

Then (24) becomes

$$\begin{array}{cc} & f(\frac{a_{o}+b_{o}}{2})\leq \int_{0}^{1}f\left(\lambda a_{o}+\left(1-\lambda \right)b_{o}\right){\left(d\lambda \right)}^{\alpha }-\int_{0}^{1}f(\frac{\left(1-2\lambda \right)|b_{o}-a_{o}|}{2}){\left(d\lambda \right)}^{\alpha }. \end{array}$$ (25)

Replacing $\lambda a_{o}+\left(1-\lambda \right)b_{o}$ by *ϰ* in (25), then we obtain

$$\begin{array}{cc} & f(\frac{a_{o}+b_{o}}{2})+\frac{1}{{\left(b_{o}-a_{o}\right)}^{\alpha }}\int_{a_{o}}^{b_{o}}f(|\frac{a_{o}+b_{o}}{2}-\varkappa |){\left(d\varkappa \right)}^{\alpha }\\ & \leq \frac{1}{{\left(b_{o}-a_{o}\right)}^{\alpha }}\int_{a_{o}}^{b_{o}}f\left(\varkappa \right){\left(d\varkappa \right)}^{\alpha }. \end{array}$$ (26)

Again as f is a generalized superquadratic then we have

$$\begin{array}{cc} & f(\lambda a_{o}+\left(1-\lambda \right)b_{o})\leq \lambda^{\alpha }(f\left(a_{o}\right)-f\left(\left(1-\lambda \right)|b_{o}-a_{o}|\right))\\ & +{\left(1-\lambda \right)}^{\alpha }(f\left(b_{o}\right)-f\left(\lambda |b_{o}-a_{o}|\right))). \end{array}$$ (27)

and

$$\begin{array}{cc} & f(\lambda b_{o}+\left(1-\lambda \right)a_{o})\leq \lambda^{\alpha }(f\left(b_{o}\right)-f\left(\left(1-\lambda \right)|b_{o}-a_{o}|\right))\\ & +{\left(1-\lambda \right)}^{\alpha }(f\left(a_{o}\right)-f\left(\lambda |b_{o}-a_{o}|\right))). \end{array}$$ (28)

Adding (27) and (28) we get

$$\begin{array}{cc} & f(\lambda a_{o}+\left(1-\lambda \right)b_{o})+f(\lambda b_{o}+\left(1-\lambda \right)a_{o})\leq f\left(a_{o}\right)+f\left(b_{o}\right)-2^{\alpha }\lambda^{\alpha }f\left(\left(1-\lambda \right)|b_{o}-a_{o}|\right))\\ & -2^{\alpha }{\left(1-\lambda \right)}^{\alpha }f\left(\lambda |b_{o}-a_{o}|\right)). \end{array}$$ (29)

If we set $\varkappa =\lambda a_{o}+\left(1-\lambda \right)b_{o}$ then $\lambda b_{o}+\left(1-\lambda \right)a_{o}=a_{o}+b_{o}-\varkappa$, $\left(1-\lambda \right)=\frac{\varkappa -a_{o}}{b_{o}-a_{o}}$ and $\lambda =\frac{b_{o}-\varkappa }{b_{o}-a_{o}}.$ So using these values in (29) we get.

$$\begin{array}{cc} & f\left(\varkappa \right)+f\left(a_{o}+b_{o}-\varkappa \right)\leq f\left(a_{o}\right)+f\left(b_{o}\right)-2^{\alpha }{\left(\frac{\varkappa -a_{o}}{b_{o}-a_{o}}\right)}^{\alpha }f\left(b_{o}-\varkappa \right)\\ & -2^{\alpha }{\left(\frac{b_{o}-\varkappa }{b_{o}-a_{o}}\right)}^{\alpha }f\left(\varkappa -a_{o}\right), \end{array}$$ (30)

now consider

$$\begin{array}{cc} & \int_{a_{o}}^{b_{o}}f\left(\varkappa \right){\left(d\varkappa \right)}^{\alpha }=\int_{a_{o}}^{\frac{a_{o}+b_{o}}{2}}f\left(\varkappa \right){\left(d\varkappa \right)}^{\alpha }+\int_{\frac{a_{o}+b_{o}}{2}}^{b_{o}}f\left(\varkappa \right){\left(d\varkappa \right)}^{\alpha }\\ & =\int_{\frac{a_{o}+b_{o}}{2}}^{b_{o}}\left(f\left(\varkappa \right)+f\left(a_{o}+b_{o}-\varkappa \right)\right){\left(d\varkappa \right)}^{\alpha }, \end{array}$$ (31)

using the value of (30) in (31), we get

$$\begin{array}{cc} & \int_{a_{o}}^{b_{o}}f\left(\varkappa \right){\left(d\varkappa \right)}^{\alpha }\leq \int_{\frac{a_{o}+b_{o}}{2}}^{b_{o}}(f\left(a_{o}\right)+f\left(b_{o}\right)-2^{\alpha }(\frac{\varkappa -a_{o}}{b_{o}-a_{o}}{)}^{\alpha }f\left(b_{o}-\varkappa \right)\\ & -2^{\alpha }(\frac{b_{o}-\varkappa }{b_{o}-a_{o}}{)}^{\alpha }f\left(\varkappa -a_{o}\right)){\left(d\varkappa \right)}^{\alpha }=\left(f\left(a_{o}\right)+f\left(b_{o}\right)\right)(\frac{{\left(b_{o}-a_{o}\right)}^{\alpha }}{2^{\alpha }})\\ & -\frac{2^{\alpha }}{{\left(b_{o}-a_{o}\right)}^{\alpha }}\int_{\frac{a_{o}+b_{o}}{2}}^{b_{o}}[{\left(\varkappa -a_{o}\right)}^{\alpha }f\left(b_{o}-\varkappa \right)+{\left(b_{o}-\varkappa \right)}^{\alpha }f\left(\varkappa -a_{o}\right)]{\left(d\varkappa \right)}^{\alpha }\\ & =\frac{f\left(a_{o}\right)+f\left(b_{o}\right)}{2^{\alpha }}{\left(b_{o}-a_{o}\right)}^{\alpha }-\frac{1}{{\left(b_{o}-a_{o}\right)}^{\alpha }}\int_{a_{o}}^{b_{o}}[{\left(\varkappa -a_{o}\right)}^{\alpha }f\left(b_{o}-\varkappa \right)+{\left(b_{o}-\varkappa \right)}^{\alpha }f\left(\varkappa -a_{o}\right)]{\left(d\varkappa \right)}^{\alpha }, \end{array}$$

it implies that

$$\begin{array}{cc} & \frac{1}{{\left(b_{o}-a_{o}\right)}^{\alpha }}\int_{a_{o}}^{b_{o}}f\left(\varkappa \right){\left(d\varkappa \right)}^{\alpha }\\ & \leq \frac{f\left(a_{o}\right)+f\left(b_{o}\right)}{2^{\alpha }}-\frac{1}{{\left(b_{o}-a_{o}\right)}^{2\alpha }}\int_{a_{o}}^{b_{o}}[{\left(\varkappa -a_{o}\right)}^{\alpha }f\left(b_{o}-\varkappa \right)+{\left(b_{o}-\varkappa \right)}^{\alpha }f\left(\varkappa -a_{o}\right)]{\left(d\varkappa \right)}^{\alpha }, \end{array}$$ (32)

from (26) and (32), we get

$$\begin{array}{cc} & f(\frac{a_{o}+b_{o}}{2})+\frac{1}{{\left(b_{o}-a_{o}\right)}^{\alpha }}\int_{a_{o}}^{b_{o}}f(|\frac{a_{o}+b_{o}}{2}-\varkappa |){\left(d\varkappa \right)}^{\alpha }\leq \frac{1}{{\left(b_{o}-a_{o}\right)}^{\alpha }}\int_{a_{o}}^{b_{o}}f\left(\varkappa \right){\left(d\varkappa \right)}^{\alpha }.\\ & \leq \frac{f\left(a_{o}\right)+f\left(b_{o}\right)}{2^{\alpha }}-\frac{1}{{\left(b_{o}-a_{o}\right)}^{2\alpha }}\int_{a_{o}}^{b_{o}}[{\left(\varkappa -a_{o}\right)}^{\alpha }f\left(b_{o}-\varkappa \right)+{\left(b_{o}-\varkappa \right)}^{\alpha }f\left(\varkappa -a_{o}\right)]{\left(d\varkappa \right)}^{\alpha }, \end{array}$$ (33)

now by setting

1(bo-ao)α∫aobof(|ao+bo2-ϰ|)(dϰ)α=Γ(1+α)(bo-ao)αIaoboαf(|ao+bo2-ϰ|),

and

1(bo-ao)α∫aobof(ϰ)(dϰ)α=Γ(1+α)(bo-ao)αIaoboαf(ϰ),

in (33) we obtain the required result.

**Remark 4** *If we put α* = 1 *then Theorem 8 will be reduced to the classical version of Hermite-Hadamard inequality*.

## Comparative analysis between fractal integral inequalities for superquadraticity and convexity

In this section, we present the comparison between the integral inequalities such that Jensen’s and Mercer Jensen’s type integral inequalities for generalised superquadratict functions and for generalised convex functions. We will see that how integral inequalities for generalised superquadratic functions are more refined than the integral inequalities for generalised convex functions. The detail of generalised Jensen,s inequality and generalised Jensen-Mercer inequality is available in [32].

**Example 7** *Let us choose Mittag-Leffler function on fractal sets* $R^{\alpha }$ (0 < *α* ≤ 1) *given by*

$$\begin{array}{c} E_{\alpha }\left(\varkappa^{\alpha }\right)=\sum_{k=0}^{\infty }\frac{\varkappa^{k\alpha }}{\Gamma (1+k\alpha )},\;\varkappa \in R, \end{array}$$

*is a generalized superquadratic function as well as generalized convex function for all ϰ* ≥ 3.

*For* $\text{n}=2,a_{o}=3,b_{o}=4,\lambda =0.2$ *and α* ∈ (0, 1], *we get the following R.H.Ss for the generalised Jensen,s inequality for superquadratic function which we denote by* R_*s*_ *and for convex function which we denote by* R_*c*_ *are as follow*.

$$\begin{array}{cc} & {\text{R}}_{s}\left(\alpha \right)=0.2^{\alpha }\left(E_{\alpha }\left(3^{\alpha }\right)-E_{\alpha }\left(0.8^{\alpha }\right)\right)+0.8^{\alpha }\left(E_{\alpha }\left(4^{\alpha }\right)-E_{\alpha }\left(0.2^{\alpha }\right)\right),\\ & {\text{R}}_{c}\left(\alpha \right)=0.2^{\alpha }\left(E_{\alpha }\left(3^{\alpha }\right)\right)+0.8^{\alpha }\left(E_{\alpha }\left(4^{\alpha }\right)\right). \end{array}$$

**Fig 11:**
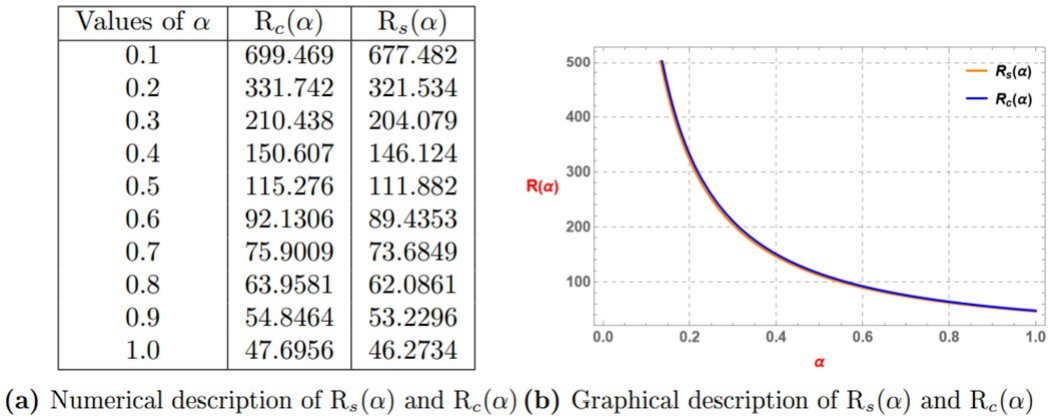
Numerical and graphical description of R_*s*_(*α*) and R_*c*_(*α*) for *α* ∈ (0, 1]. (**a**) Numerical description of R_*s*_(*α*) and R_*c*_(*α*). (**b**) Graphical description of R_*s*_(*α*) and R_*c*_(*α*).

*Consider the subsequent Table 11a and Graph 11b given by* Fig 11, *which display that generalized superquadratic function provides refined Jensen’s inequality than the generalized convex function*.

**Example 8** *Let us consider the same function as in Example 7 For* $\text{n}=2,a_{o}=3,b_{o}=4,\lambda =0.2$ *and α* ∈ (0, 1], *we get the following R.H.Ss for the generalised Mercer Jensen’s inequality for superquadratic function which we denote by* R_*s*_ *and for convex function which we denote by* R_*c*_ *are as follow*.

$$\begin{array}{cc} & {\text{R}}_{s}\left(\alpha \right)=E_{\alpha }\left(3^{\alpha }\right)+E_{\alpha }\left(4^{\alpha }\right)-0.2^{\alpha }\left(E_{\alpha }\left(3^{\alpha }\right)+E_{\alpha }\left(0.8^{\alpha }\right)\right)-0.8^{\alpha }\left(E_{\alpha }\left(4^{\alpha }\right)+E_{\alpha }\left(0.2^{\alpha }\right)\right)\\ & {\text{R}}_{c}\left(\alpha \right)=E_{\alpha }\left(3^{\alpha }\right)+E_{\alpha }\left(4^{\alpha }\right)-0.2^{\alpha }\left(E_{\alpha }\left(3^{\alpha }\right)\right)-0.8^{\alpha }\left(E_{\alpha }\left(4^{\alpha }\right)\right). \end{array}$$

**Fig 12:**
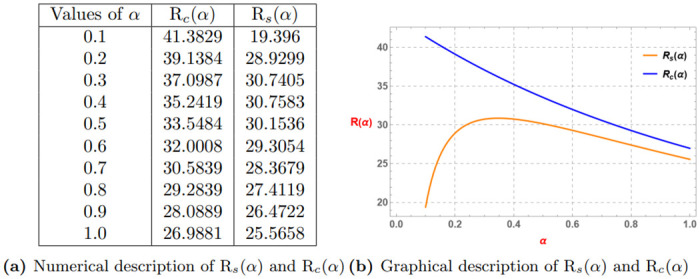
Numerical and graphical description of R_*s*_(*α*) and R_*c*_(*α*) for *α* ∈ (0, 1]. (**a**) Numerical description of R_*s*_(*α*) and R_*c*_(*α*). (**b**) Graphical description of R_*s*_(*α*) and R_*c*_(*α*).

*Consider the subsequent Table 12a and Graph 12b given by* Fig 12, *which display that generalized superquadratic function provides refined Mercer Jensen’s inequality than the generalized convex function*.

## Applications

### 0.1 Probabilistic characteristics

Let we have a random variable X and its expectation and variance in the fractal sense is designated by *E*_*α*_[X] and *D*_*α*_[X] respectively and given as follows such that.

$$\begin{array}{c} E_{\alpha }\left[\text{X}\right]=\Sigma_{i=1}^{n}\lambda_{i}^{\alpha }\varkappa_{i}, \end{array}$$ (34)

and

$$\begin{array}{c} D_{\alpha }^{2}\left[\text{X}\right]=E_{\alpha }\left[{\text{X}}^{2}\right]-{\left(E_{\alpha }\left[\text{X}\right]\right)}^{2}, \end{array}$$

we shall perpetually presuppose that all random variables have real valued and are not degenerate, as well as their expectations are available. One of the most common and fundamental inequalities in probability theory in the fractal space is outlined as below:

$$\begin{array}{c} E_{\alpha }\left[f\left(\text{X}\right)\right]\geq f\left(E\left[\text{X}\right]\right), \end{array}$$ (35)

where f is supposed to be the generalised convex function. The expression (35) is obtained by using (34) in the following generalised Jensen’s inequality.

$$\begin{array}{cc} & \text{f}(\Sigma_{i=1}^{n}\lambda_{i}\varkappa_{i})\leq \Sigma_{i=1}^{n}\lambda_{i}^{\alpha }\text{f}(\varkappa_{i}). \end{array}$$

Let I ⊂ R be an interval. We have the following probabilistic characterization of generalized superquadraticity.

**Theorem 9** *The function* f: **I** → R^*α*^ *is a generalized superquadratic if*

$$\begin{array}{cc} & \text{f}(E[\text{X}])\leq E_{\alpha }\left[f\left(\text{X}\right)\right]-E_{\alpha }\left[f\left(\text{X}-E\left[\text{X}\right]\right)\right]. \end{array}$$ (36)

***proof***: *The result is obtained by taking into account the Theorem 5 which by* (35), *is equivalent to* (36).

**Theorem 10** *The function* $f:I\subset \text{R}\to {\text{R}}^{\alpha }$ *be a generalized superquadratic function for* (m_*o*_, M_*o*_) ⊆ *I and for all* X ∈ (m_*o*_, M_*o*_) *if*

$$\begin{array}{cc} & E_{\alpha }\left[f\left(\text{X}\right)\right]\leq (\frac{{\text{M}}_{o}^{\alpha }-E_{\alpha }\left[\text{X}\right]}{{\text{M}}_{o}^{\alpha }-{\text{m}}_{o}^{\alpha }})[f\left({\text{m}}_{o}\right)-f\left(\text{X}-{\text{m}}_{o}\right)]+(\frac{E_{\alpha }\left[\text{X}\right]-{\text{m}}_{o}^{\alpha }}{{\text{M}}_{o}^{\alpha }-{\text{m}}_{o}^{\alpha }})(f\left({\text{M}}_{o}\right)-f\left({\text{M}}_{o}-\text{X}\right)). \end{array}$$

**proof**: *By Theorem 6 the function* $f:I\subset \text{R}\to {\text{R}}^{\alpha }$ *be a generalized superquadratic function for* (m_*o*_, M_*o*_) ⊆ *I and for all* X ∈ (m_*o*_, M_*o*_) *if it satisfies the inequality* (16), *and then keeping in view* (35) *we attain*

$$\begin{array}{cc} & E_{\alpha }\left[f\left(\text{X}\right)\right]\leq [(\frac{{\text{M}}_{o}^{\alpha }-E_{\alpha }\left[\text{X}\right]}{{\text{M}}_{o}^{\alpha }-{\text{m}}_{o}^{\alpha }})f\left({\text{m}}_{o}\right)+(\frac{E_{\alpha }\left[\text{X}\right]-{\text{m}}_{o}^{\alpha }}{{\text{M}}_{o}^{\alpha }-{\text{m}}_{o}^{\alpha }})f\left({\text{M}}_{o}\right)]\\ & -[(\frac{{\text{M}}_{o}^{\alpha }-E_{\alpha }\left[\text{X}\right]}{{\text{M}}_{o}^{\alpha }-{\text{m}}_{o}^{\alpha }})f\left(\text{X}-{\text{m}}_{o}\right)+(\frac{E_{\alpha }\left[\text{X}\right]-{\text{m}}_{o}^{\alpha }}{{\text{M}}_{o}^{\alpha }-{\text{m}}_{o}^{\alpha }})f\left({\text{M}}_{o}-\text{X}\right)]\\ & =(\frac{{\text{M}}_{o}^{\alpha }-E_{\alpha }\left[\text{X}\right]}{{\text{M}}_{o}^{\alpha }-{\text{m}}_{o}^{\alpha }})[f\left({\text{m}}_{o}\right)-f\left(\text{X}-{\text{m}}_{o}\right)]+(\frac{E_{\alpha }\left[\text{X}\right]-{\text{m}}_{o}^{\alpha }}{{\text{M}}_{o}^{\alpha }-{\text{m}}_{o}^{\alpha }})(f\left({\text{M}}_{o}\right)-f\left({\text{M}}_{o}-\text{X}\right)) \end{array}$$

### 0.2 Special means

Let $a_{o}<b_{o}$ and $a_{o},b_{o}\in \text{R}$, considering the following *α*-type special means.

(1) The generalized arithmetic mean:

$$\begin{array}{c} {\text{A}}_{\alpha }\left(a_{o},b_{o}\right)=\frac{{a_{o}}^{\alpha }+{b_{o}}^{\alpha }}{2^{\alpha }}. \end{array}$$

(2) The generalized p-logarithmic mean:

$$\begin{array}{c} {\text{L}}_{p\alpha }\left(a_{o},b_{o}\right)={\left[\frac{\Gamma (1+p\alpha )}{\Gamma (1+(p+1)\alpha )}\frac{{b_{o}}^{(p+1)\alpha }-{a_{o}}^{(p+1)\alpha }}{{\left(b_{o}-a_{o}\right)}^{\alpha }}\right]}^{\frac{1}{p}}, \end{array}$$

Where p∈Z\{-1,0}, $a_{o},b_{o}\in \text{R}$, $a_{o}\neq b_{o}$

(3) The generalized geometric mean:

$$\begin{array}{c} {\text{G}}_{\alpha }\left(a_{o},b_{o}\right)=\sqrt{{a_{o}}^{\alpha }{b_{o}}^{\alpha }}. \end{array}$$

Consider the mapping $f:\left[1,\infty \right)\to {\text{R}}^{\alpha },f\left(\varkappa \right)=\frac{\Gamma (1+p\alpha )}{\Gamma (1+(p+1)\alpha )}\varkappa^{(p+1)\alpha }$, ϰ>0,p≥2 is a generalised superquadratic function on [1, ∞).

**Proposition 3** *For* $a_{o},b_{o}\in \left[1,\infty \right)$ , $0<a_{o}<b_{o}$ *and* p≥2 *we have*

$$\begin{array}{c} ({\text{A}}_{\alpha }\left(a_{o},b_{o}\right){)}^{p+1}\leq {\text{A}}_{\alpha }\left({a_{o}}^{p+1},{b_{o}}^{p+1}\right)-({\text{A}}_{\alpha }\left(-a_{o},b_{o}\right){)}^{p+1}. \end{array}$$ (37)

**proof**: *The result can be proved by using the function* $f\left(\varkappa \right)=\frac{\Gamma (1+p\alpha )}{\Gamma (1+(p+1)\alpha )}\varkappa^{(p+1)\alpha }$ *in Theorem 5 for n* = 2, *and taking* $\lambda_{1}=\lambda_{2}=\frac{1}{2}$.

To verify the reliability of Proposition 3, we take into consideration the next example.

**Example 9** *We attain the subsequent Right and Left terms of the* (37) for *α* = 0.4 *and* p=2.

$$\begin{array}{cc} & Left\;\;\;\;Term=({\text{A}}_{0.4}\left(a_{o},b_{o}\right){)}^{3}\\ & Right\;\;\;\;Term={\text{A}}_{0.4}\left({a_{o}}^{3},{b_{o}}^{3}\right)-({\text{A}}_{0.4}\left(-a_{o},b_{o}\right){)}^{3}. \end{array}$$

**Fig 13:**
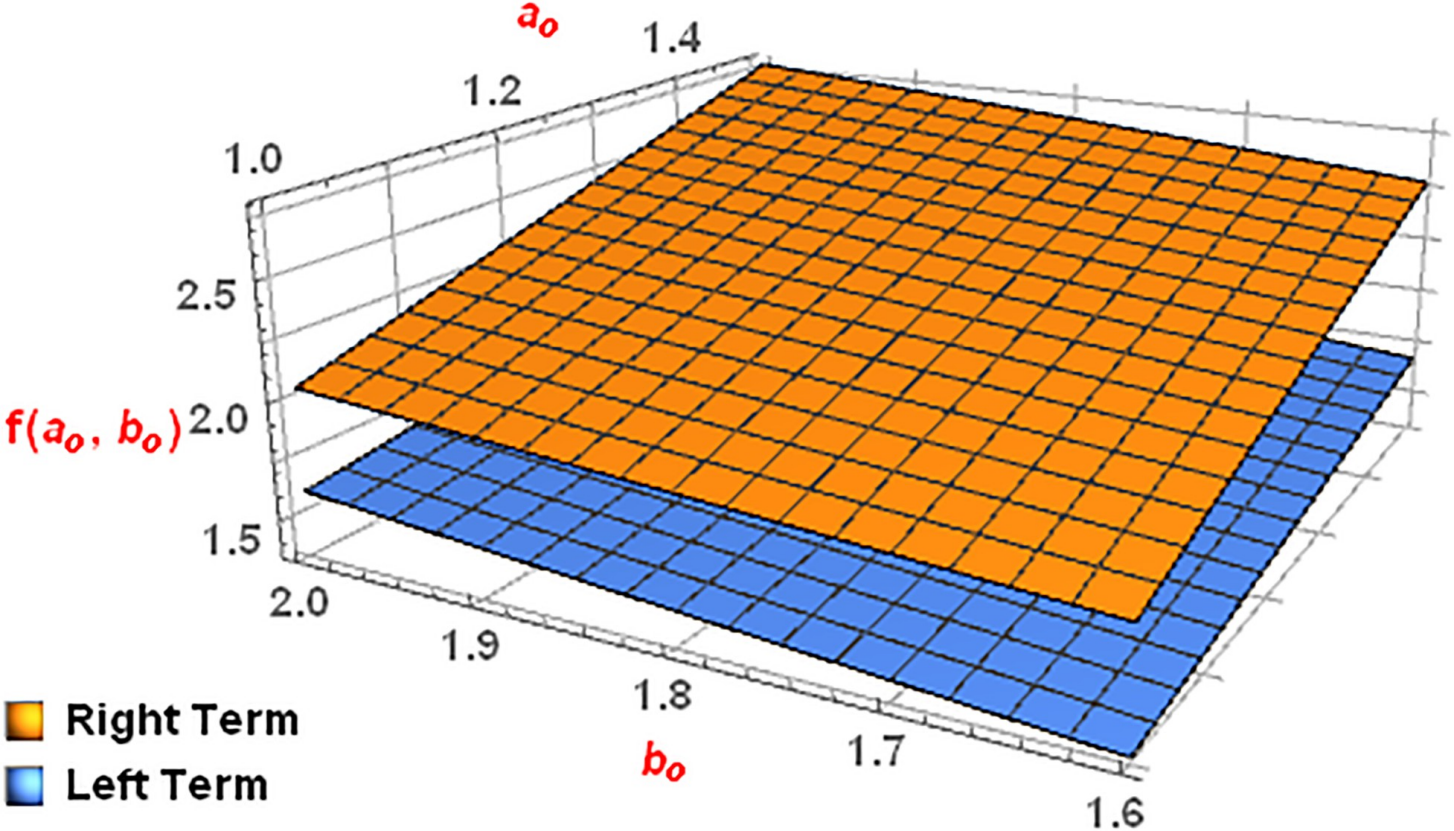
Graphical illustration of Right and Left terms for $a_{o}\in [1,1.5]$ and $b_{o}\in [1.6,2]$.

*Now for* $a_{o},b_{o}\in [1,2]$ *we have the below mentioned 3D* Fig 13, *for the Right and Left terms*.

*Hence the aforementioned* Fig 13 *confirms the validity of Proposition 3*.

## Conclusion

In this research, we have introduced the concept of generalized superquadratic functions on fractal sets. By defining these functions, we have analyzed their unique features and determined their significant inequalities. We have derived several important inequalities for generalized superquadratic functions. Our study offers a detailed comparative analysis of the inequalities for generalized superquadratic functions versus those for generalized convex functions. This comparison has highlighted the greater refinement and applicability of the inequalities derived from generalized superquadratic functions, showcasing their added scientific value. We have demonstrated the practical relevance of our findings through applications in probability expectations and special means in fractal space. These applications validate our theoretical results and illustrate their potential utility in various applied domains. While our study has made significant contributions, it has focused primarily on a specific class of generalized superquadratic functions. In future, one can explore further inequalities and properties of other generalized superquadratic functions, such as generalized h-superquadratic, s-superquadratic, P-superquadratic, and multiplicative superquadratic functions. By applying the methods used in this paper to other classes of functions and inequalities to gain new insights and extend the theoretical framework. Our research extends the existing literature by introducing and analyzing generalized superquadratic functions on fractal sets. The findings contribute significantly to the field of mathematical inequalities and have the potential to inspire further studies and applications in various scientific and engineering disciplines. This work underscores the scientific value added by our study and highlights the broad applicability of our results.
